# Fast and accurate inference of gene regulatory networks through robust precision matrix estimation

**DOI:** 10.1093/bioinformatics/btac178

**Published:** 2022-03-23

**Authors:** Antoine Passemiers, Yves Moreau, Daniele Raimondi

**Affiliations:** ESAT-STADIUS, KU Leuven, 3001 Leuven, Belgium; ESAT-STADIUS, KU Leuven, 3001 Leuven, Belgium; ESAT-STADIUS, KU Leuven, 3001 Leuven, Belgium

## Abstract

**Motivation:**

Transcriptional regulation mechanisms allow cells to adapt and respond to external stimuli by altering gene expression. The possible cell transcriptional states are determined by the underlying gene regulatory network (GRN), and reliably inferring such network would be invaluable to understand biological processes and disease progression.

**Results:**

In this article, we present a novel method for the inference of GRNs, called PORTIA, which is based on robust precision matrix estimation, and we show that it positively compares with state-of-the-art methods while being orders of magnitude faster. We extensively validated PORTIA using the DREAM and MERLIN+P datasets as benchmarks. In addition, we propose a novel scoring metric that builds on graph-theoretical concepts.

**Availability and implementation:**

The code and instructions for data acquisition and full reproduction of our results are available at https://github.com/AntoinePassemiers/PORTIA-Manuscript. PORTIA is available on PyPI as a Python package (portia-grn).

**Supplementary information:**

Supplementary data are available at *Bioinformatics* online.

## 1 Introduction

Transcriptional regulation is a crucial mechanism that allows cells to adapt to changing environmental conditions and respond to external stimuli (Sławek and Arodź, 2013) by dynamically modulating their gene expression. Transcription factors (TFs) play a central role in this behaviour by regulating their own expression and the one of their downstream target genes (TGs) (Aibar *et al.*, 2017), effectively constituting complex gene regulatory networks (GRNs) that underlie the possible transcriptional states of each cell (Aibar *et al.*, 2017). Although gene expression is also impacted by higher-level epigenomic regulation mechanisms (chromatin accessibility and DNA methylation) (Klemm *et al.*, 2019), TFs have the most relevant role. For this reason, elucidating the structure of GRNs is crucial for understanding both physiological cell processes and pathological mechanisms (Ruyssinck *et al.*, 2014). A deeper understanding of GRNs could indeed open possibilities for the treatment of complex diseases such as cancer (Plaisier *et al.*, 2016; Chen *et al.*, 2014).

Inferring GRNs from experimental gene expression data is a non-trivial challenge that poses several major issues due to the noisiness, scarcity and complexity of the available data (Gardner and Faith, 2005) which cause the severe under-determination of the problem (Ruyssinck *et al.*, 2014). Moreover, gene expression data are heterogeneous, since they can be acquired from different experimental methods, such as (i) wild-type measurement (naturally occuring expression levels), (ii) time series (multiple observations obtained after initial perturbation of the system), (iii) multifactorial perturbation data (simultaneous perturbation of multiple genes), or from more controlled experiments such as (iv) transient gene knock-downs (KD) and (v) homozygous gene knock-outs (KO). In the first case, a gene’s expression is reduced through genetic techniques performed at RNA level (e.g. RNAi). In knock-out experiments, both copies of the same gene are made non-functional (e.g. through non-sense mutations or null mutations that result in a complete loss-of-function). Each type of experiment leads to data with certain peculiarities. For example, time series allow causal analysis of GRNs (Geurts *et al.*, 2018), but are scarce and can suffer from design issues such as the need for cell synchronization over time (Bar-Joseph *et al.*, 2012). KO experiments are very informative as they show how the intervention on a gene affects the rest of the network. The causal relationship is captured using the so-called null-mutant *Z*-score (Prill *et al.*, 2010). However, such approach requires knocking out each gene separately, making the whole procedure time-consuming and expensive. KD experiments are less controlled than the latter, since interventions are often performed at *transcriptional level*. Finally, multifactorial data are the least expensive, as they allow the study of multiple regulatory genes in parallel, but are less informative than data collected through other techniques. KO, KD and multifactorial data are all steady-state measurements obtained after initial perturbation of the system.

Because of its complexity, the analysis of this data requires algorithmic methods. Various computational methods for the reconstruction of GRNs have been proposed during the last decades. They can roughly be categorized as follows: logical models, continuous models, information-theoretic approaches and feature selection methods based on machine learning models.

Logical models build upon Boolean functions to model the relation between genes (genes are either expressed or unexpressed), and include Boolean probabilistic and stochastic networks (Kauffman, 1969; Shmulevich *et al.*, 2002; Liang and Han, 2012) as well as Petri nets (Heiner *et al.*, 2012). Because discrete values are not suited for modelling subtle variations in gene expression values, methods including linear models (D’haeseleer *et al.*, 1999; Gardner *et al.*, 2003; Yip *et al.*, 2010), Bayesian networks (Friedman *et al.*, 2000; Perrin *et al.*, 2003; Grzegorczyk and Husmeier, 2011), linear and non-linear autoregressive models (Michailidis and d’Alché Buc, 2013), as well as models based on differential equations (Chen *et al.*, 1999; Kikuchi *et al.*, 2003) have been proposed.

Correlations and mutual information (Stuart *et al.*, 2003; Steuer *et al.*, 2002) have been used to quantify the statistical dependence between genes, but predicting the GRN structure from correlation matrices directly leads to the prediction of a large amount of false positives. Therefore, more sophisticated approaches based on conditional mutual information (Zhang *et al.*, 2012) or on the maximum relevance/minimum redundancy principle (Meyer *et al.*, 2007) were proposed. ARACNe (Margolin *et al.*, 2006a,b) and ARACNe-AP (Lachmann *et al.*, 2016) rely on the data processing inequality to discard spurious correlations from its predictions.

More recently, feature selection approaches based on Machine Learning methods such as Random Forest (Breiman, 2001) or gradient boosting (Friedman, 2001) have been used. GENIE3 (Irrthum *et al.*, 2010) and its time-series-targeted variant dynGENIE3 (Geurts *et al.*, 2018) build on the former, while ENNET (Sławek and Arodź, 2013) and GRNBoost (Aibar *et al.*, 2017) on the latter. PLSNET (Guo *et al.*, 2016) and TIGRESS (Haury *et al.*, 2012) rely instead on partial least squares and least angle regression, respectively. Finally, NIMEFI (Ruyssinck *et al.*, 2014) generalizes the task of feature selection by combining multiple models, like random forests, elasticnet and support vector machines.

In this article, we describe PORTIA, a novel algorithm for GRN inference based on power transforms and covariance matrix inversion. In our vision, a key aspect of GRN inference is the need to disentangle direct from indirect (e.g. transitive) correlations. Our work has thus been conceptually inspired by Direct Coupling Analysis methods used in the field of protein contact prediction (Jones *et al.*, 2012; Baldassi *et al.*, 2014), but several major adaptations were necessary to transfer these concepts to the GRN inference task.

We benchmarked PORTIA on the widely used DREAM datasets, as well as the more recent MERLIN+P datasets, showing that it competes very well with state-of-the-art models, while being orders of magnitude faster. We also analysed the potential causes of its mispredictions, showing that beyond performance, the topology of GRNs inferred by PORTIA differs from other methods. Finally, we propose a novel and more informative scoring metric for the GRN inference task, based on these graph-theoretical concepts. PORTIA is freely available at: https://github.com/AntoinePassemiers/PORTIA.

## 2 Materials and methods

### 2.1 Datasets

We benchmarked our method on GRN inference datasets provided in the editions 3, 4 and 5 of the DREAM (Dialogue for Reverse Engineering Assessments and Methods) challenge. All datasets are available on the website https://www.synapse.org/ and are widely used in literature as common benchmark within the GRN inference community. In addition, we evaluated our method on the more recent MERLIN-P datasets (Siahpirani and Roy, 2017).

From DREAM3, we considered the *In Silico Size 100* dataset, and we refer to it as DREAM3 for short. From DREAM4, we considered the *In Silico Size 100* (we call it DREAM4 from now on) and the *In Silico Size 100 Multifactorial* (called DREAM4MF) datasets. Each of these datasets contains five different *in silico* gene networks generated with GeneNetWeaver (Schaffter *et al.*, 2011). Except for DREAM4MF, the datasets comprise time series, gene KO and gene KD experiments.

From the DREAM5 challenge, we adopted the *Network Inference Challenge* dataset (called DREAM5 from now on), composed of one *in silico* network generated with GeneNetWaver, as well as two *in vivo* networks from *Escherichia* *coli.* and *Saccharomyces* *cerevisiae*. Despite being two well-studied organisms, the drawback of evaluating on these *real* networks is the incompleteness of the experimentally determined regulatory interactions. Inferred networks are being evaluated on these verified interactions, even though they might constitute a subset of the actual network. DREAM5 consists of a combination of multifactorial data, sparse time series and very few KO experiments. It is worthy of note that DREAM5 is the only DREAM challenge for which a list of potential TFs was provided. Network sizes, as well as the number of measurements and other statistics, are provided in Supplementary Table S1.

We further validated PORTIA on datasets from the MERLIN+P study. Three types of yeast expression datasets are considered: natural variation (NatVar), knock-out (KO) and response to stress (StressResp). Each of the GRNs inferred from these datasets was evaluated with three different goldstandard networks (Siahpirani and Roy, 2017). Finally, performance on two natural variation datasets from human lymphoblastoid cell lines (LCL) was assessed, based on the same goldstandard network derived from regulator perturbation data (Cusanovich *et al.*, 2014).

### 2.2 PORTIA: a method for Gaussian modelling of GRNs

**Fig. 1.**
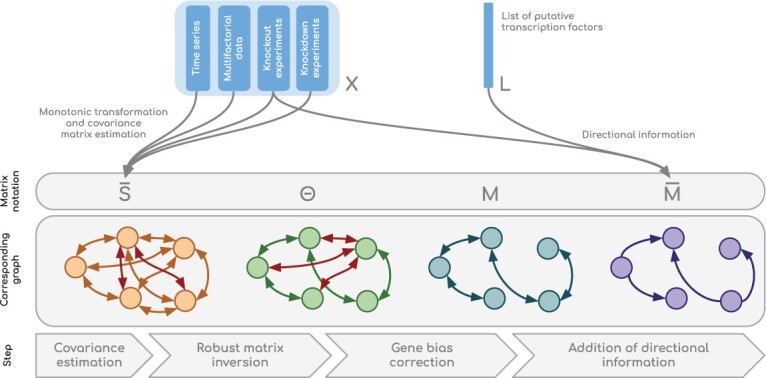
Illustrative summary of our methods. All gene expression data (multifactorial, time series, KO, KD, etc.) are concatenated as a single matrix *X* used to estimate a covariance matrix $\bar{S}$. The latter is ensured to be full-rank and its inverse is denoted by Θ. A correction step is performed to filter out gene-specific biases. Finally, directional information is added in order to predict an asymmetric score matrix $\bar{M}$. The adjacency matrix of the reconstructed network is obtained by setting a threshold for the scores in $\bar{M}$

In this section, we describe PORTIA, our algorithm for GRN inference. The full pipeline is shown in Figure 1. We represent each target dataset as a gene expression matrix $X\in R_{+}^{n\times m}$ composed of *n* expression measurements and *m* genes. For the DREAM5 and MERLIN+P datasets, a list *L* of potential TFs is provided. If such a list is not available, we consider by default that every gene *j* is in *L*. This is equivalent to assuming that the GRN can be *any* sub-graph of the complete graph.

Gene expression levels generally have an asymmetric (skewed) distribution, and are governed by non-linear regulatory relationships. For this reason, Gaussian modelling might not be suitable, unless a prior non-linear transformation of the data is performed beforehand. We thus processed the input data *X* by applying a power transform on each gene (each column) individually, ensuring that the relationship between gene expressions becomes more linear, and the joint distribution more Gaussian-like. Namely, we assume that the relationship between the expression of two genes *i* and *j* before processing is non-linear *but* monotonic [i.e. a positive change in the expression of a TF always induces a positive (negative) change in the expression of a TG if the sign of the regulatory relationship is positive (negative), and vice versa.]. More specifically, each column $X_{\cdot j}$ is transformed using the Box–Cox transform (Box and Cox, 1964), a general-purpose (monotonic) power transform:

(1) $$Y_{ij}=\{\begin{array}{cc} \frac{X_{ij}^{\lambda_{j}}-1}{\lambda_{j}}\; & \text{if}\;\lambda_{j}\neq 0\\ \;\text{log}\;\;X_{ij}\; & \text{otherwise} \end{array}$$

where *λ* is a parameter vector found by maximum likelihood estimation. Power transforms are usually applied on each column of the data matrix *X* independently. However, enforcing the marginals to be Gaussian does not guarantee that the joint distribution will be Gaussian too. For this reason, we propose an extension of our approach, called etePORTIA, that jointly optimizes the parameters of the power transforms. We described it in Supplementary Material.

*In vivo* data usually contain more genes than experimental measurements and thus the sample covariance matrix *S* is likely to be rank-deficient. To overcome this problem, we perform a shrinkage estimation of the covariance matrix. Let $D=I⊙S\in R^{m\times m}$ be a diagonal matrix, where *I* is the identity matrix and ⊙ the Hadamard product. Consequently, *D_jj_* corresponds to the variance in the expression levels of gene *j*. The shrinkage (Ledoit and Wolf, 2004) estimator $\bar{S}=\alpha I⊙S+(1-\alpha )S$ is a convex linear combination of sparse matrix *D* and sample covariance matrix *S*. When $\bar{S}$ is well-conditioned (with *α* sufficiently large), its inverse Θ (see end-result of step 2 in Fig. 1) can be accurately estimated. We did not infer *α* using the approach from Ledoit–Wolf (Ledoit and Wolf, 2004), since our goal is to minimize quadratic risk on the precision matrix instead of the covariance matrix. We used 0.8 in all of our experiments, a value that is much greater than all *α* estimations found with the Ledoit–Wolf method (<0.3 on the DREAM datasets).

As next step (third step in Fig. 1), we corrected the precision matrix in order to remove gene-specific biases caused by the small sample size. The idea was pioneered in the context of protein contact prediction and has been shown to be crucial for accurate reconstruction of protein contact maps. This step also turned out to play a determining role in our own methodological developments. Techniques include average product correction (Dunn *et al.*, 2008) or additive row-column weighting (Gouveia-Oliveira and Pedersen, 2007). We did not perform average product correction, to preserve the interpretation of conditional independence in Gaussian Graphical Models ($\Theta_{i,j}=0\Leftrightarrow y_{i}\;⫫\;\;y_{j}|\{y_{k}\;\forall k\notin \{i,j\}\}$): each zero entry in Θ should remain zero after correction. Instead, we performed a multiplicative row-column weighting. Also, impossible regulatory links were removed: a gene cannot regulate another gene if it is not listed in *L* among the potential transcription factors. Let $\mu (\Theta_{i\cdot })$ be the average of non-zero values in column $\Theta_{i\cdot }$. Rows corresponding to genes that are not in *L* were discarded during the computations of $\Theta_{\cdot j}$. The corrected matrix *M* is thus computed as follows:

(2) $$M_{ij}=\{\begin{array}{cc} \frac{2|\Theta_{ij}|}{\mu (|\Theta_{i\cdot }|)\mu (|\Theta_{\cdot j}|)}\;\; & \;\;\text{if}\;i\in L\\ 0\;\; & \;\;\text{otherwise} \end{array}$$

### 2.3 Modelling edge directionality

Due to the undirected nature of Gaussian Graphical Models, the approach described in the previous section will *a priori* not perform well unless it is supplemented with directional information. For this reason, we considered an additional step (fourth step in Fig. 1), using three sources of directional information that do not impact the speed of the whole inference process, namely the expression data itself, the list of potential TFs and null-mutant *Z*-scores computed from KO experiments. However, all these information might not necessarily be available in all contexts.

First, we re-weighted each score *M_ij_* with weight $B_{ij}/\text{max}(B_{ij},B_{ji})$, where *B_ij_* is the *i*th coefficient of linear regression for the prediction of the *j*th gene’s expression. We propose a way to solve the *m* linear regression problems in quadratic time, and provide the details in Supplementary Material.

In Equation 2, the relation between TF *i* and TG *j* is discarded if i∉L (where *L* is the list of TFs). Sidelining of such relations poses an issue only if undiscovered TFs are missing from *L*. Precisely to limit this risk in the framework of the DREAM5 challenge, sensitivity was privileged over specificity in the TF selection process. Assuming there are no false negatives among potential TFs, *L* is a great source of directional hints, as *x*% of regulatory links can be discarded when *L* contains 1−x% of the total network size.

Another way of taking into account the directionality of regulatory relationships, is to rely on null-mutant *Z*-scores, similarly to the method described in Greenfield *et al.* (2010). We propose to compute these scores as follows:

(3) $$Z_{ij}=\{\begin{array}{cc} \frac{\left|X_{ij}^{\mathrm{knockout}}-\mu_{j}\right|}{\sigma_{j}}\;\; & \;\;\text{if KOs available for genes}\;i\;\text{and}\;j\\ Z\;\; & \;\;\text{otherwise} \end{array}$$

where Z is the median of all *Z*-scores across all KO experiments, $X_{i\cdot }^{\mathrm{knockout}}$ are the expression levels for KO experiment of gene *i*, *μ_j_* is the average expression level of TG *j* across all KO experiments, and *σ_j_* the standard deviation obtained similarly. We discarded experiments involving multiple knocked-out genes, to avoid dealing with the ambiguity caused by these genes being confounders. When multiple KO experiments are available for the same TF, then *Z*-scores are averaged across experiments. Calculation of these *Z*-scores is a principled way of approximating the causal effect of a gene KO on all other genes, since it quantifies the deviation of the expression of all genes (but one) after KO from the background noise. Since *Z*-scores, when they can be computed, are highly informative about causal relationships in GRNs, we will also report their performance as a standalone baseline method in the Section 3.

The final score matrix $\bar{M}$ predicted by PORTIA is obtained as follows: ${\bar{M}}_{ij}=M_{ij}Z_{ij}^{2}$. We squared the elements in *Z* to attach a higher degree of importance to the top scores derived from interventional data. For low values in *Z*, interventional and observational data have approximately equal importance. Because squaring is a scale-dependent operation, *Z* is first *L*^2^-normalized.

Also, we performed a post-processing step on $\bar{M}$, where each row is multiplied by its standard deviation. This step is of great importance as it allows high scores to stand out of the background noise. Indeed, because GRNs are likely to be scale-free networks, where most genes are regulated by a few hubs (Liu and Hu, 2019), detecting these hubs would enable the accurate prediction of many regulatory links at once. Therefore, we used standard deviation as a proxy for the node centrality of these *hub* genes.

Finally, the sign of each regulatory link can be simply reported as the sign of the corresponding element in the precision matrix. Indeed, because genes are assumed to be co-expressed in a monotonic manner, their expression correlates either positively or negatively.

### 2.4 Performance assessment

We used the DREAMTools Python package (Cokelaer *et al.*, 2015) to compare reconstructed networks to the goldstandard networks from DREAM3 and DREAM4. With regard to DREAM5, reconstructed networks were evaluated with the help of the MATLAB script provided on the competition’s website. Finally, we measured the performance on the MERLIN+P datasets ourselves (the code is available on our repository). For each dataset, the same overall score metric was computed as follows (Marbach *et al.*, 2012):

(4) $$\text{Overall score}=-\frac{1}{2}{\;\text{log}\;}_{10}({\bar{p}}_{\mathrm{AUROC}}\;{\bar{p}}_{\mathrm{AUPR}})$$

where ${\bar{p}}_{\mathrm{AUROC}}$ and ${\bar{p}}_{\mathrm{AUPR}}$ are *P*-values obtained after computing the AUROC and AUPR scores on 25 000 randomly generated networks.

Inferred networks are directed (in the sense that the direction of the regulatory link is predicted), but evaluated regardless of the mode of regulation, meaning that no consideration is given to whether a TF enhances or inhibits the expression of regulated genes. We remind that, however, PORTIA natively provides such information.

## 3 Results

In the following sections, we show the results of our method PORTIA and its variant etePORTIA against state-of-the-art GRN inference methods on four widely used datasets (see Methods) from past editions of the DREAM challenge, as well as the MERLIN+P datasets.

### 3.1 PORTIA is the best performing on all DREAM datasets except DREAM4MF

Table 1 shows the performance of PORTIA on the DREAM3 dataset. etePORTIA has *both* higher AUPR and AUROC than any other method on network 1, remains highly competitive for the other networks, and has the highest overall score. Since single-gene KO experiments are provided in an exhaustive manner for both DREAM3 and DREAM4, we reported also the performance of null-mutant *Z*-scores described in Section 2.3 (which are already part of PORTIA’s pipeline) as a separate baseline method for these datasets. Interestingly, PORTIA and its end-to-end version barely improve the baseline *Z*-score method on DREAM3. It might be suggested that performance on this dataset is independent of the degree of sophistication of the modelling, but rather mostly related to the appropriate use of interventional data (ENNET, PORTIA and etePORTIA, are all three based on KO-derived *Z*-scores).

**Table 1.** AUROC, AUPR and overall scores of different GRN inference methods, evaluated on the five networks from DREAM3

Method	Net1		Net2		Net3		Net4		Net5		Overall score (no KO)	Overall score
	AUPR	AUROC	AUPR	AUROC	AUPR	AUROC	AUPR	AUROC	AUPR	AUROC		
ARACNe-AP	0.021	0.563	0.030	0.555	0.039	0.581	0.056	0.530	0.065	0.513	2.475	2.975
GENIE3	0.019	0.602	0.014	0.552	0.021	0.532	0.037	0.491	0.060	0.514	0.574	1.289
PLSNET	0.018	0.541	0.029	0.526	0.044	0.674	0.065	0.576	0.071	0.517	2.742	4.835
TIGRESS	0.050	0.760	0.051	0.692	0.045	0.628	0.066	0.562	0.071	0.526	8.128	8.151
ENNET	0.382	0.887	0.593	0.926	0.347	0.866	0.273	0.770	0.236	0.684	5.372	78.413
*Z*-scores	0.692	0.913	0.854	0.963	0.576	0.887	0.508	0.847	0.445	0.788	—	142.938
PORTIA	0.726	0.956	0.826	0.986	0.512	0.888	0.507	0.873	0.385	0.798	3.492	144.029
etePORTIA	0.728	0.956	0.832	0.986	0.516	0.888	0.506	0.872	0.386	0.798	3.598	144.373

DREAM4 results shown in Table 2 are similar to DREAM3, as *Z*-scores alone exhibit good performance. However, we notice a significant improvement of PORTIA and etePORTIA with respect to *Z*-scores (+28.956 and +29.4, respectively). Overall, both versions of PORTIA outperform all other approaches. Also, PORTIA and etePORTIA have higher AUPRs and AUROCs than the state-of-the-art on three out the five networks, while ENNET leads on the remaining two ones. After the removal of KO experiments from the datasets in DREAM3 and DREAM4, all methods are underperforming as shown in the second-to-last column in Tables 1 and 2, also revealing how some methods *implicitly* benefit from interventional data (e.g. PLSNET, but mostly GENIE3). In both cases, TIGRESS is performing the best (with 8.128 and 24.873 scores, respectively).

**Table 2.** AUROC, AUPR and overall scores of different GRN inference methods, evaluated on the five networks from DREAM4

Method	Net1		Net2		Net3		Net4		Net5		Overall score (no KO)	Overall score
	AUPR	AUROC	AUPR	AUROC	AUPR	AUROC	AUPR	AUROC	AUPR	AUROC		
ARACNe-AP	0.052	0.614	0.073	0.601	0.096	0.630	0.063	0.614	0.080	0.650	10.086	10.934
GENIE3	0.105	0.835	0.101	0.766	0.182	0.821	0.113	0.807	0.128	0.821	1.840	32.307
PLSNET	0.055	0.765	0.058	0.704	0.083	0.740	0.073	0.746	0.059	0.712	10.046	17.057
TIGRESS	0.090	0.807	0.072	0.695	0.162	0.797	0.099	0.748	0.107	0.765	24.873	24.723
ENNET	0.462	0.894	0.384	0.853	0.455	0.880	0.418	0.867	0.312	0.853	23.886	80.753
*Z*-scores	0.407	0.898	0.357	0.806	0.383	0.818	0.318	0.838	0.141	0.769	—	64.296
PORTIA	0.613	0.932	0.504	0.890	0.438	0.869	0.472	0.888	0.292	0.840	13.271	93.252
etePORTIA	0.619	0.935	0.514	0.889	0.437	0.869	0.462	0.889	0.286	0.846	14.418	93.696

**Fig. 2.**
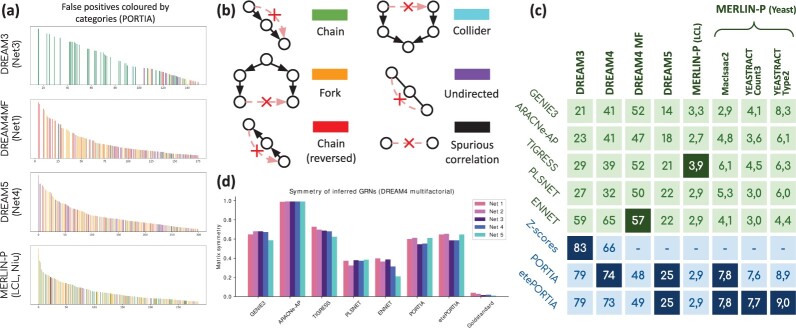
(**a**) Top scores predicted by PORTIA on networks from four different datasets. *X*-axis is the ranking of the gene pair, *Y*-axis is the score of the gene pair (in log-scale) and the presence of a bar at position *i* indicates that reporting the corresponding *i*th pair as a regulatory link would result in a FP, and its colour indicates the causal structure of the sub-network wherein the FP occurs, as illustrated in (**b**). (b) Colour legend for (a). (**c**) Average normalized discounted cumulative gain (NDCG) of each method on each dataset, measured in percentage. (**d**) Matrix symmetry of the inferred and goldstandard networks from the DREAM4MF dataset

Table 3 reports the performance of each method on DREAM4MF. Because of the absence of directional information (e.g. TF list), and despite the theoretical ability of PORTIA to infer asymmetric adjacency matrices, our method only outperforms ARACNe-AP. On this dataset, GRN reconstruction performance seem to strongly reflect the asymmetry of inferred GRNs, as shown in Figure 2d. ENNET outperforms all other methods on three out of the five networks, and has the highest overall score (52.543). Overall, all methods have similar results, except ARACNe-AP which produced the lowest score (17.520).

**Table 3.** AUROC, AUPR and overall scores of different GRN inference methods, evaluated on the five networks proposed in the DREAM4 *in silico* network challenge, size 100 multifactorial networks

Method	Net1		Net2		Net3		Net4		Net5		Overall score
	AUPR	AUROC	AUPR	AUROC	AUPR	AUROC	AUPR	AUROC	AUPR	AUROC	
ARACNe-AP	0.119	0.602	0.086	0.568	0.163	0.655	0.131	0.645	0.124	0.627	17.520
GENIE3	0.156	0.750	0.153	0.726	0.229	0.764	0.217	0.788	0.191	0.795	37.008
PLSNET	0.110	0.716	0.265	0.828	0.227	0.796	0.208	0.819	0.186	0.780	44.155
TIGRESS	0.159	0.751	0.156	0.698	0.228	0.765	0.214	0.779	0.224	0.755	36.426
ENNET	0.179	0.725	0.262	0.802	0.287	0.811	0.296	0.821	0.282	0.831	52.543
PORTIA	0.137	0.693	0.139	0.706	0.230	0.773	0.229	0.778	0.144	0.725	32.819
etePORTIA	0.138	0.706	0.151	0.704	0.237	0.774	0.230	0.778	0.155	0.729	34.050

Table 4 summarizes results on the three networks of DREAM5. Overall, PORTIA and etePORTIA outperform all methods on the *in vivo E.coli* and *S.cerevisiae* networks, which constitue the hardest problem instances. ENNET has the highest overall score (infinity) due to an abnormally high contribution from the *in silico* network (which mostly has to do with the way *P*-values are produced, based on stretched exponentials). Results remain similar after the removal of KO experiments.

**Table 4.** AUROC, AUPR and overall scores of different GRN inference methods, evaluated on the three networks proposed in the DREAM5 GRN inference sub-challenge

Method	*In silico*		*E.coli*		*S.cerevisiae*		Overall score (no KO)	Overall score
	AUPR	AUROC	AUPR	AUROC	AUPR	AUROC		
ARACNe-AP	0.174	0.682	0.056	0.566	0.020	0.516	0.418	1.723
GENIE3	0.288	0.812	0.096	0.620	0.021	0.518	0.000	39.304
PLSNET	0.238	0.853	0.043	0.569	0.020	0.514	34.251	37.972
TIGRESS	0.307	0.781	0.067	0.592	0.020	0.514	33.914	31.803
ENNET	0.438	0.848	0.054	0.608	0.019	0.512	65.948	>300
PORTIA	0.383	0.822	0.110	0.620	0.028	0.537	41.691	75.425
etePORTIA	0.385	0.822	0.110	0.620	0.028	0.536	43.143	76.374

### 3.2 PORTIA positively compares with other methods for the reconstruction of GRNs from yeast and human lymphoblastoid cell lines

Each multi-column reported in Table 5 corresponds to a separate gene expression dataset. For the yeast datasets, metrics have been averaged across the three available goldstandard networks. PORTIA and etePORTIA have the highest overall scores (45.852 and 45.891, respectively), and are only outperformed by TIGRESS on the goldstandard network from Geuvadis for lymphoblastoid cell lines (LCL), where TIGRESS is the only method producing networks better than random. It must be noted that the strong differences between Niu’s and Geuvadis’ goldstandards in terms of performance can very likely be attributed to the high sparsity and little agreement between these networks (the underlying experiments involve different TFs). Regardless, PORTIA is the only method that systematically produced significant results on the three yeast datasets for each goldstandard network (*P*-value < 0.01). AUROC, AUPR and *P*-value are provided for each goldstandard network in Supplementary Tables S9–S12. We attribute the overall low performance on these datasets to the strong sparsity of goldstandard networks, giving little room for evaluating the most confident predictions of each method, and the inherent difficulty of the task: *in vivo* networks have complex underlying mechanisms, and these mechanisms may be idealized when generating GRNs artificially (e.g. DREAM). However, results on the MERLIN+P and DREAM5 suggest that PORTIA is able to provide more accurate reconstructions of GRNs when the number of involved genes is very large.

**Table 5.** ROC-AUC scores of different GRN inference methods on three yeast expression datasets and an LCL dataset from MERLIN-P, evaluated on three and two goldstandard networks, respectively

Method	LCL (Niu)		LCL (Geuvadis)		NatVar (Average)		KO (Average)		Stress (Average)		Overall score
	AUPR	AUROC	AUPR	AUROC	AUPR	AUROC	AUPR	AUROC	AUPR	AUROC	
ARACNe-AP	0.137	0.503	0.134	0.493	0.034	0.578	0.019	0.521	0.022	0.548	3.687
GENIE3	0.125	0.482	0.137	0.501	0.015	0.481	0.016	0.506	0.016	0.502	0.323
PLSNET	0.130	0.484	0.118	0.468	0.033	0.523	0.015	0.488	0.019	0.514	14.977
TIGRESS	0.138	0.500	0.150	0.520	0.020	0.498	0.020	0.520	0.015	0.497	1.587
ENNET	0.128	0.491	0.128	0.483	0.037	0.569	0.024	0.521	0.028	0.536	17.463
PORTIA	0.140	0.502	0.141	0.502	0.110	0.657	0.029	0.552	0.031	0.559	45.852
etePORTIA	0.140	0.509	0.140	0.505	0.111	0.660	0.028	0.552	0.031	0.559	45.891

### 3.3 PORTIA is orders of magnitude faster than existing methods

Supplementary Tables S2 and S3 report the complexity and running times of different GRN inference methods, including ours. All computations were performed on an AMD EPYC processor with 16 cores and 64 GB RAM (CentOS 8). Despite its cubic complexity (when *L* contains all *m* genes), it clearly appears that PORTIA is orders of magnitude faster than other state-of-the-art methods. As expected, the end-to-end version etePORTIA is comparatively slower since the estimated covariance matrix has to be factorized at each iteration until convergence is reached.

## 4 Discussion

### 4.1 A novel GRN inference evaluation metric based on the underlying causal structures

The hypothesis behind PORTIA’s development is that GRN inference methods need to reliably filter out indirect correlations from predicted relations, analogously to direct coupling-based protein contact prediction methods (Dunn *et al.*, 2008; Jones *et al.*, 2012). However, standard metrics like AUROC or AUPR are not sufficient to fully characterize to what extent the disentanglement of direct and indirect correlations occurs, especially when computed on the whole gene network. Therefore, we looked at the causes of mispredictions from a graph-theoretic perspective.

For each network, we categorised the false-positive (FP) cases according to the causal structure of the sub-network wherein the regulatory link is predicted. Next, we associated a relevance score with each category and computed a metric reflecting the overall relevance of the inferred network. In graphs illustrated in Figure 2b, plain arrows correspond to existing relations in the goldstandard and dashed arrows marked with a cross correspond to regulatory relations that are (erroneously) present in the inferred network. We grouped all possible cases into categories, sorted by decreasing order of relevance:

- *True positive*: A gene directly regulates another gene.
- *Chain*: A gene indirectly regulates another gene.
- *D-connected genes* that fall in none of the two previous categories: The two genes are either part of a *fork* (they are indirectly regulated by the same TF) or a reversed chain (indirect regulatory relation predicted in the wrong direction)
- *D-separated genes*: This category is composed of *colliders* (2 TFs regulating the same gene) and undirected links (remaining cases). Two genes *A* and *B* are d-separated if there is no TF *C* regulating both of them.
- *Spurious, etc.*: No indirect causal relationship can justify the presence of a FP. We refer to *spurious correlations* as correlations that cannot be attributed to anything causal, including indirect effects (forks, chains, etc.), regardless of the directionality of regulatory links in the goldstandard networks. However, it is likely that many FPs will fall in this category within *in vivo* networks (networks from DREAM5 and MERLIN+P) due to the fact that our knowledge of these networks is still incomplete.

We propose a variant of the Normalized Discounted Cumulative Gain (NDCG), taking into account the relevance of each prediction, as an evaluation metric for quantifying the information content of a reconstructed GRN. Details about its implementation are provided in Supplementary Materials. A ready-to-use Python implementation of NDCG is available as part of the portia-grn Python package. NDCG scores, averaged across all networks in a same dataset, are reported in Figure 2c for each dataset. It can be noted that PORTIA and its end-to-end variant etePORTIA outperform other methods on DREAM3 (except the baseline *Z*-scores), DREAM4, DREAM5 and all yeast goldstandard networks from MERLIN+P.

### 4.2 The causes of misprediction are dataset-dependent

Beyond NDCG scores, finer analysis reveals the different difficulties that GRN inference tools are facing. The causal structures wherein false positive (FPs) occur not only depend on the inference tool itself, but also on the dataset, as shown in Figure 2a for PORTIA. Each bar indicates a FP among the top-scoring predictions, and its colour relates to the causal structure (given by panel b of the same figure) that is the most likely to justify the presence of such FP. On DREAM3, most of the top-scoring gene pairs resulting in FPs occur in regulatory chains. Indeed, the incorporation of interventional data allows PORTIA to discard confounding effects and mostly report meaningful causal relationships. However, KO experiments do not allow to disentangle direct causal relationships from chains. Such bar plot is not shown for DREAM4, as it strongly resembles what has been observed for DREAM3. The dataset on which PORTIA produces the highest proportion of reversed chains (genes at the ends of a regulatory chain inferred in the wrong direction) is DREAM4MF, revealing the difficulty of the method at inferring the correct direction of regulatory links, even among its highest-scoring pairs. Overall, a large proportion of FPs is explained by the presence of forks. Indeed, two genes that are d-connected are expected to show a correlation, which can be attributed to a common TF. Causal structures are less consistent on the MERLIN+P datasets, which is mostly due to the strong sparsity of the experimentally verified interactions. The FP counts for each causal structure, GRN inference method and network have been reported in Supplementary Tables S4–S8.

### 4.3 Interventional data better contribute to the accurate reconstruction of GRNs than observational data

Causal relationships cannot be inferred from observations alone, and require either assumptions or additional information collected through interventions (Pearl, 2009). From the perspective of causal calculus, gene KO experiments are valuable interventions as they allow sampling from $P(X|\;\text{do}(X_{\cdot j}=0))$, and null-mutant *Z*-scores are simple approximations of how dissimilar this distribution is from *P*(*X*). Expressions containing the do(·) operator are used to formalize causal relationships, but they can be evaluated only with the aid of experimental interventions, such as null mutations (complete loss-of-function of a gene). In particular, $\text{do}(X_{\cdot j}=0))$ refers to gene *j* not being expressed due to a KO mutation.

To empirically show the relevance of KO data, we removed it from the datasets, when applicable. Overall scores were reported for DREAM3, DREAM4 and DREAM5 as an extra column in Tables 1, 2 and 4, respectively. In addition, full performance comparison with AUPRs and AUROCs is shown in Supplementary Tables S13–S15. A strong loss of performance was noted for PORTIA, etePORTIA, GENIE3 and ENNET on DREAM3 and DREAM4. Surprisingly, we even observed a loss of 44.7% of the overall score on DREAM5 (from 75.425 to 41.691) for PORTIA, despite the fact that single-gene KOs were given for only 1.1% of the genes on average. The significant performance drop of ENNET can also be attributed to its modelling, as it also relies on *Z*-scores. What is even more striking is the catastrophic performance loss of GENIE3 (from 39.304 to 0), notwithstanding the absence of explicit modelling of KO data. GENIE3 exhibits the same behaviour on DREAM3 (1.289–0.574) and DREAM4 (32.307–1.840). This shows the importance of interventional data in the discovery of causal relationships, even when such data are sparse, and even when their modelling is implicit.

Finally, a slight improvement of TIGRESS can be systematically observed after removal of KO data, from 8.151 to 8.128 on DREAM3, from 24.723 to 24.873 on DREAM4 and from 31.803 to 33.914 on DREAM5. TIGRESS outperforms PORTIA in such settings on DREAM3 and DREAM4, however, this poses questions about the scalability of its accuracy with respect to the availability of interventional data. Indeed, the performance of GRN inference tools can reasonably be expected to scale with the elucidation of real networks.

## 5 Conclusion

In this article, we presented PORTIA, a fast and accurate tool devised for inferring GRNs from heterogeneous gene expression datasets. Our method positively compares with state-of-the-art approaches, while being at least one order of magnitude faster. In addition, we proposed a novel scoring metric for the evaluation of inferred GRNs, based on the local causal structures in the goldstandard networks, thus re-weighting false positives based on their severity. This metric, which is a variant of the normalized discounted cumulative gain, better captures the directionality and levels of indirection of predicted regulatory relationships than general-purpose metrics like AUROC or AUPR. Finally, we highlight the explicit (e.g. ENNET, PORTIA) and implicit (e.g. GENIE3, PLSNET) dependence of GRN inference tools on KO experiments, suggesting that the performance of some methods (e.g. GENIE3) is not solely driven by the sophistication of their modelling.

## Supplementary Material

btac178_Supplementary_Data
