# Supplementary material for: Fast and accurate inference of gene regulatory networks through robust precision matrix estimation
Source: Bioinformatics. 2022 Mar 23;38(10):2802–9. doi: 10.1093/bioinformatics/btac178 (PMC9113237; doi:10.1093/bioinformatics/btac178)
Supplement: btac178_Supplementary_Data [file btac178_supplementary_data.pdf]

# Supplementary materials

March 31, 2022

## Contents

**S1 Datasets** **2**

**S2 CPU benchmark** **3**

**S3 Methodology** **4**
S3.1 End-to-end inference . . . . . . . . . . . . . . . . . . . . . . . . . . . . 4
S3.2 Solving $m$ regression problems from a single precision matrix . . . . . . . 6
S3.2.1 Problem statement . . . . . . . . . . . . . . . . . . . . . . . . . 6
S3.2.2 Removing the effect of the target gene . . . . . . . . . . . . . . . . 6
S3.2.3 Improving the directionality of inferred GRNs . . . . . . . . . . . . 7

**S4 Computational efficiency** **8**
S4.1 Incomplete precision matrix estimation . . . . . . . . . . . . . . . . . . . 8
S4.2 Cholesky factorisation during end-to-end inference . . . . . . . . . . . . . 8
S4.3 The Box-Cox transform is only suitable for strictly positive values . . . . 8

**S5 Performance assessment** **9**
S5.1 Performance metrics . . . . . . . . . . . . . . . . . . . . . . . . . . . . . 9
S5.1.1 Overall score . . . . . . . . . . . . . . . . . . . . . . . . . . . . 9
S5.1.2 Normalised discounted cumulative gain . . . . . . . . . . . . . . . 10
S5.1.3 Matrix symmetry . . . . . . . . . . . . . . . . . . . . . . . . . . 11

**S6 Results** **17**
S6.1 Performance on MERLIN+P datasets . . . . . . . . . . . . . . . . . . . . 17
S6.2 Effect of KO removal . . . . . . . . . . . . . . . . . . . . . . . . . . . . 19
S6.3 Symmetry of inferred adjacency matrices . . . . . . . . . . . . . . . . . . 20

**S7 Future work** **24**

# S1 Datasets

We summarise in Table S1 general statistics about the datasets used for validating PORTIA. These statistics include the network size (number of genes), the number of measurements, the number of (experimentally) verified interactions, the number of single gene knock-out experiments and the size of the TF whilelist $L$, if provided. When multiple knock-out experiments are available for the some knocked-out gene, the latter is reported only once. Dual, triple and quadruple knock-out experiments are not accounted at all, since they are less informative than single knock-outs due to knocked-out genes confounding each others.

Table S1: Summary of the different goldstandard GRNs.

| Dataset | Network | Organism | Genes | Samples | Interactions | Knock-outs | $\|L\|$ |
|---|---|---|---|---|---|---|---|
| D3 | Net1 | in silico | 100 | 1168 | 125 | 100 | - |
| | Net2 | in silico | 100 | 1168 | 119 | 100 | - |
| | Net3 | in silico | 100 | 1168 | 166 | 100 | - |
| | Net4 | in silico | 100 | 1168 | 389 | 100 | - |
| | Net5 | in silico | 100 | 1168 | 551 | 100 | - |
| D4 | Net1 | in silico | 100 | 411 | 176 | 100 | - |
| | Net2 | in silico | 100 | 411 | 249 | 100 | - |
| | Net3 | in silico | 100 | 411 | 195 | 100 | - |
| | Net4 | in silico | 100 | 411 | 211 | 100 | - |
| | Net5 | in silico | 100 | 411 | 193 | 100 | - |
| D4MF | Net1 | in silico | 100 | 100 | 176 | 0 | - |
| | Net2 | in silico | 100 | 100 | 249 | 0 | - |
| | Net3 | in silico | 100 | 100 | 195 | 0 | - |
| | Net4 | in silico | 100 | 100 | 211 | 0 | - |
| | Net5 | in silico | 100 | 100 | 193 | 0 | - |
| D5 | Net1 | in silico | 1643 | 805 | 4012 | 35 | 195 |
| | Net3 | E. Coli | 4511 | 805 | 2066 | 39 | 334 |
| | Net4 | S. Cerevisiae | 5950 | 536 | 3940 | 13 | 333 |
| MERLIN+P (MacIsaac2) | NatVar | Yeast | 1939 | 377 | 3786 | 0 | 261 |
| | KO | Yeast | 1943 | 404 | 3795 | - | 259 |
| | StressResp | Yeast | 1924 | 173 | 3756 | 0 | 260 |
| MERLIN+P (YEASTRACT Count 3) | NatVar | Yeast | 2118 | 377 | 3801 | 0 | 246 |
| | KO | Yeast | 2117 | 404 | 3786 | - | 245 |
| | StressResp | Yeast | 2151 | 173 | 3841 | 0 | 246 |
| MERLIN+P (YEASTRACT Type 2) | NatVar | Yeast | 2205 | 377 | 4201 | 0 | 247 |
| | KO | Yeast | 2206 | 404 | 4183 | - | 246 |
| | StressResp | Yeast | 2255 | 173 | 4257 | 0 | 247 |
| MERLIN+P (LCL) | Niu | H. Sapiens | 2761 | 287 | 6383 | 0 | 368 |
| | Geuvadis | H. Sapiens | 2761 | 462 | 6383 | 0 | 368 |

DREAM3 and DREAM4 networks, as well as the *in silico* network of the DREAM5 challenge, were created with GeneNetWeaver [14]. GeneNetWeaver generates graph topologies by extracting known structures from organisms such as *E. Coli* and *S. cerevisiae*. These structures are broken down into so-called *modules*, which are sets of strongly associated genes. At the gene level, mRNA and protein concentrations are modelled each by a differential equation. A chemical Langevin equation is added to model the noisy fluctuations caused by translational and transcriptional processes. mRNA concentration is determined by a translation and a degradation term, and translation is itself a function

of TF concentrations. Whether a TF is an enhancer or an inhibitor is determined from extracted modules.

There are 9 rows reported for the MERLIN+P yeast datasets. However, these rows come from 3 yeast expression datasets and 3 goldstandard networks. Because each dataset has been used to infer networks that were further evaluated against 3 goldstandard networks, there are indeed 9 evaluations in total. The 3 yeast expression datasets are natural variation data (NatVar), knock-out experiments (KO) and response to stress (StressResp). The first dataset (NatVar) is a concatenation of gene expression from [2, ], [15, ] and [16, ], while Knockout is composed of datasets from [4, ] and [8, ], and StressResp comprises data from [8, ].

Lymphoblastoid cell lines (LCL) consist of 2 datasets of natural variation data from [10, ] and [12, ]. Inferred LCL GRNs were scored against a goldstandard network from Geuvadis [6, ].

# S2 CPU benchmark

Running times of the different GRN inference tools are reported in tables S2 and . All computations were performed on an AMD EPYC processor with 16 cores and 64 GB RAM (CentOS 8).

Table S2: CPU times of different GRN inference methods (in seconds) on DREAM datasets. $n$, $m$ and $q$ are the number of observations, genes and TFs, respectively. $t$ corresponds to the number of trees in GENIE3, the number of bootstrap runs in ARACNE-AP, and the number of iterations in ENNET, PLSNET and etePORTIA. In TIGRESS, $R$ is the number of resampling runs (which was set to 4000 for small networks) and $L$ the number of iterations of the least-angle regression algorithm.

| Method | Complexity | Parameters | DREAM 3 | DREAM 4 | DREAM 4M | DREAM 5 | | |
|---|---|---|---|---|---|---|---|---|
| | | | Average | Average | Average | Net1 | Net3 | Net4 |
| GENIE3 | $\mathcal{O}(tm\sqrt{q}n\log(n))$ | $t = 1000$, 4 threads | 252 | 80 | 32 | 2901 | 9030 | 7820 |
| TIGRESS | $\mathcal{O}(nmqRL)$ | $L = 5, R \in \{4000, 1000\}$ | 533 | 280 | 195 | 4810 | 5375 | 4430 |
| ARACNE-AP | $\mathcal{O}(tm^3)$ | $t = 100$ | 256 | 95 | 38 | 1254 | 6535 | 7044 |
| PLSNET | $\mathcal{O}(tm\sqrt{m}n)$ | $t = 1000$ | 212 | 120 | 68 | 4399 | 10870 | 10650 |
| ENNET | $\mathcal{O}(tm^2n)$ | $t = 5000$ | 157 | 57 | 15 | 3453 | 16665 | 14288 |
| PORTIA | $\mathcal{O}(m^3)$ | - | $<$ **1** | $<$ **1** | $<$ **1** | **2** | **8** | **12** |
| etePORTIA | $\mathcal{O}(tm^3)$ | $t = 100$ | 162 | 158 | 84 | 648 | 2481 | 3144 |

Table S3: CPU times of different GRN inference methods (in seconds) on MERLIN+P datasets. Each multi-column corresponds to a different goldstandard network, while each single column corresponds to a gene expression dataset.

| Method | Yeast | | | | | | | | | LCL | |
|---|---|---|---|---|---|---|---|---|---|---|---|
| | MacIsaac2 | | | YEASTRACT Count 3 | | | YEASTRACT Type 2 | | | Niu | Geuvadis |
| | NatVar | KO | StressResp | NatVar | KO | StressResp | NatVar | KO | StressResp | | |
| GENIE3 | 1590 | 1773 | 583 | 1719 | 1967 | 633 | 1864 | 1993 | 666 | 1817 | 3369 |
| TIGRESS | 1672 | 1085 | 713 | 1171 | 1137 | 771 | 1255 | 1212 | 793 | 3270 | 4569 |
| ARACNe-AP | 1401 | 1330 | 677 | 1537 | 1386 | 719 | 1685 | 1514 | 728 | 1597 | 2475 |
| PLSNET | 980 | 1014 | 632 | 1055 | 1081 | 736 | 1104 | 1131 | 764 | 1124 | 1458 |
| ENNET | 2474 | 2926 | 1277 | 2807 | 3029 | 1348 | 2989 | 3122 | 1427 | 4109 | 6639 |
| PORTIA | **18** | **17** | **15** | **18** | **18** | **16** | **18** | **17** | **18** | **20** | **20** |
| etePORTIA | 1269 | 1093 | 998 | 1426 | 1218 | 1109 | 1423 | 1231 | 1151 | 1676 | 2060 |

# S3 Methodology

## S3.1 End-to-end inference

In the present section, we provide the mathematical formulation of the optimisation problem underlying etePORTIA, our end-to-end version of PORTIA.

As a reminder, the Box-Cox transform is defined by:

$$Y_{ij} = \begin{cases} \dfrac{X_{ij}^{\lambda_j} - 1}{\lambda_j} & \text{if } \lambda_j \neq 0 \\ \log X_{ij} & \text{otherwise} \end{cases} \tag{1}$$

where $\lambda$ is a parameter vector found by maximum likelihood estimation. In order for the Gaussian probability density function (p.d.f.) to preserve its properties, and for the likelihood to be comparable across different values of $Y_{\cdot j}$ during optimization, we have to multiply it by the Jacobian $|\mathbb{J}|$ of the power transform. The log-likelihood is thus of the following form [1, ]:

$$\ell(\lambda_j) = -\frac{1}{2} n \log \sigma^2(Y_{\cdot j}) + \log |\mathbb{J}| \tag{2}$$

where $\sigma^2(Y_{\cdot j})$ is the variance of the $j$-th column of $Y$.

The Jacobian is defined as the determinant of the Jacobian matrix. Because observations are independent and independently transformed, the Jacobian matrix is diagonal. Therefore, its determinant is simply the product of its diagonal entries. For a given gene $j$, it is thus computed as follows:

$$\log |\mathbb{J}| = \log \prod_{i=1}^{n} \frac{\partial Y_{ij}}{\partial X_{ij}} = \sum_{i=1}^{n} \log \frac{\partial}{\partial X_{ij}} \frac{X_{ij}^{\lambda_j} - 1}{\lambda_j} = (\lambda_j - 1) \sum_{i=1}^{n} \log X_{ij} \tag{3}$$

$Y \in \mathbb{R}^{n \times m}$ denotes the transformed data matrix where each column results from the maximum likelihood estimation for that column. According to our monotonicity assumption, observations in $Y$ follow a multivariate Gaussian distribution. The corresponding sample

covariance $S \in \mathbb{R}^{m \times m}$ is directly estimated from the transformed data $Y$. Finally, the shrinkage estimator of the covariance matrix is given by $\bar{S} = \alpha I \odot S + (1 - \alpha)S$.

Let us also remember the negative log-likelihood of the multivariate Gaussian distribution:

$$\min_\Theta \quad \frac{1}{2}(n-1)\text{tr}(S\Theta) - \frac{1}{2}n \ \text{logdet}(\Theta) \tag{4}$$

$$\text{s.t.} \quad \Theta \text{ is } s.p.d. \tag{5}$$

Because $S$ is not necessarily invertible, or is not necessarily well-conditioned, the objective function is often penalized by a L1 regularisation term like in the Graphical Lasso approach [7, ]. Instead of enforcing sparsity, we propose to comply with the sequential version of PORTIA and replace the L1 regularisation by shrinkage estimation. Also, in order to improve the normality of gene expression data, we propose to jointly optimise the parameters of the power transforms. We refer to this end-to-end inference approach as etePORTIA.

More specifically, we want to find the parameter vector $\lambda \in \mathbb{R}^m$ that maximizes the likelihood function in equation 6. For the log-likelihood function to be comparable across different values of $\lambda$, one needs to take into account the non-linear transformations by multiplying the likelihood by the Jacobian of the transformation. The maximum likelihood estimator for the corresponding precision matrix is the one that solves the following problem:

$$\min_\Theta \quad \frac{1}{2}(n-1)\text{tr}(S\Theta) - \frac{1}{2}n \ \text{logdet}(\Theta) - \log \mathbb{J} \tag{6}$$

$$\text{s.t.} \quad \Theta \text{ is } s.p.d. \tag{7}$$

where $\log \mathbb{J}$ is the log-determinant of the Jacobian matrix associated with the power transform, and $\Theta$ is a symmetric positive-definite matrix. Covariance matrix shrinkage of $S$ as above is invertible when $\alpha$ is sufficiently high. The final optimization problem is the following:

$$\min_\lambda \quad \frac{1}{2}(n-1)\text{tr}(S\Theta) - \frac{1}{2}n \ \log\ \det(\Theta) - \log\ \mathbb{J} \tag{8}$$

$$\text{where} \quad \begin{cases} Y_{ij} = \dfrac{X_{ij}^{\lambda_j} - 1}{\lambda_j} \\ \log \mathbb{J} = \displaystyle\sum_{j=1}^{m} (\lambda_j - 1) \sum_{i=1}^{n} \log(X_{ij}) \\ \Theta = \big(\alpha I \odot S + (1-\alpha)S\big)^{-1} \end{cases} \tag{9}$$

Due to the non-convexity of the problem, we opted for the software library PyTorch [13, ] to perform inference, through gradient-based optimization. All mathematical operations described in our methods are differentiable, including matrix inversion. Parameters are iteratively updated by the Adam optimizer [9, ], using a learning rate of 0.05, and decay rates of 0.9 and 0.999 for the moving averages.

## S3.2 Solving $m$ regression problems from a single precision matrix

### S3.2.1 Problem statement

GRN inference can be expressed as a feature selection problem. This can be achieved by fitting one linear regression per target gene, and reporting feature importances as the likelihood of each TF regulating this target gene. More formally, we define $B \in \mathbb{R}^{m \times m}$ as the matrix of feature importances, where $B_{\cdot j}$ is the ordinary least squares (OLS) estimator of the parameters of the following linear regression:

$$X_{\cdot j} = B_{1,j} X_{\cdot 1} + \ldots + B_{j-1,j} X_{\cdot j-1} + B_{j+1,j} X_{\cdot j+1} + \ldots + B_{m,j} X_{\cdot m} \tag{10}$$

As a consequence of the Gauss-Markov theorem, the OLS estimator for such regression is simply $(X_{(j)}^T X_{(j)})^{-1} X_{(j)}^T X_{\cdot j}$, where $X_{\cdot j}$ is the $j$-th column of $X$, and $X_{(j)}$ is the expression matrix $X$ with the $j$-th column removed. For notational convenience, we replace $X_{(j)}^T X_{(j)}$ by the shrinkage estimator of the sample covariance sub-matrix $S_{(j)}$ (the covariance matrix of all variables but the $j$-th one). After replacement, the estimator becomes:

$$B_{\cdot j} = c\big(\alpha I + (1-\alpha) S_{(j)}\big)^{-1} X_{(j)}^T X_{\cdot j} = c \bar{S}_{(j)}^{-1} X_{(j)}^T X_{\cdot j} \tag{11}$$

This is equivalent, up to a constant factor $c$, to what is obtained by Tikhonov regularisation. Because $c$ only depends on the number of genes and the shrinkage parameter, and therefore is the same across all regression problem instances, it can be safely discarded.

### S3.2.2 Removing the effect of the target gene

However, computing this estimator for each target gene directly would result in a $\mathcal{O}(m^4)$ complexity, which is not scalable. We propose to solve the $m$ regression problems with $\mathcal{O}(m^3)$ operations instead. We draw the attention of the reader on the fact that the sub-matrix $\bar{S}_{(j)}$ is the overall shrinkage estimator $\bar{S}$ with row $j$ and column $j$ removed. Therefore, we aim at cancelling the contribution of gene $j$ to the precision matrix by zeroing row $j$ and column $j$ in $\bar{S}$. In order to avoid recomputing the inverse matrix, we only estimate the effect on the precision matrix directly.

Let $u$ be a binary vector with all zeros except component $j$, and $v$ be $-\bar{S}_{\cdot j}^T = -\bar{S}_{j\cdot}$. Zeroing the $j$-th row and column is done by performing the following operations sequentially:

$$((\bar{S} + uv^T)^T + uv^T)^T \tag{12}$$

Let's note that the $j$-th diagonal element of the resulting matrix is non-zero (it has become $-\bar{S}_{jj}$), thus preserving its invertibility. To completely remove the effect of gene $j$, we finally have to zero this diagonal element at the level of the precision matrix instead. Let $J_{(j)}$ be the identity matrix $I \in \{0,1\}^{m \times m}$ with the $j$-th diagonal element set to zero. The final estimator is then given by:

$$B_{\cdot j} = \big(((\bar{S} + uv^T)^T + uv^T)^T\big)^{-1} J_{(j)} X^T X_{\cdot j} \tag{13}$$

The inverse of $\bar{S} + uv^T$ can be computed efficiently, and is given by the Sherman-Morrison formula:

$$\big(\bar{S} + uv^T\big)^{-1} = \bar{S}^{-1} - \frac{\bar{S}^{-1} u v^T \bar{S}^{-1}}{1 + v^T \bar{S}^{-1} u} \tag{14}$$

The precision matrix $\bar{S}^{-1}$ is already known, as it is needed by PORTIA to infer the GRN.

For notational convenience, we define $W = (\bar{S}+uv^T)^T$. Let's now remove the $j$-th column from $W$:

$$\begin{aligned}
\big((W + uv^T)^T\big)^{-1} &= \big(W + uv^T\big)^{-1} \\
&= W^{-1} - \frac{W^{-1}uv^T W^{-1}}{1 + v^T W^{-1} u} \\
&= (S^{-1})^T - \Big(\frac{\bar{S}^{-1}uv^T\bar{S}^{-1}}{1 + v^T\bar{S}^{-1}u}\Big)^T - \frac{W^{-1}uv^T W^{-1}}{1 + v^T W^{-1} u}
\end{aligned} \tag{15}$$

Plugging this into equation 13, the OLS estimator becomes:

$$B_{\cdot j} = (S^{-1})^T J_{(j)} X^T X_{\cdot j} - \Big(\frac{\bar{S}^{-1}uv^T\bar{S}^{-1}}{1 + v^T\bar{S}^{-1}u}\Big)^T J_{(j)} X^T X_{\cdot j} - \frac{W^{-1}uv^T W^{-1}}{1 + v^T W^{-1} u} J_{(j)} X^T X_{\cdot j} \tag{16}$$

The estimation of column $B_{\cdot j}$ still requires matrix multiplications, which have cubic complexity. Because there are $m$ columns, this still results in an overall quartic (4-th order) complexity. However, these operations are shared by all target genes, and jointly computing the $m$ columns can result in a loss of one order of complexity. After tedious computations that are not shown for the sake of brevity, the joint estimation of $B$ is performed according to algorithm 1.

**Algorithm 1** Fast algorithm for solving the $m$ linear regression problems

**Require:** Design matrix $X \in \mathbb{R}_+^{n \times m}$
**Ensure:** $B$ is the weighted adjacency matrix of the linear Structural Equation Model

```
S ← α₂I + (1 − α₂)((1/(n−1)) XᵀX)          ▷ Shrinkage estimator
T ← S⁻¹                                     ▷ Precision matrix
B ← TXᵀX
V ← −S − I
W ← VᵀT
d ← 1/(1+eᵀVT)
B ← B − T repeat(d ⊙ (eᵀ((XW) ⊙ X)))
K ← T − W repeat(diag(T) ⊙ d)
f ← eᵀ(W ⊙ V)
q ← eᵀ(XK ⊙ X)
B ← B − T repeat((eᵀWV) ⊙ d ⊙ q/(1+eᵀVK))
```

In the pseudo-code, $\alpha_2$ is the shrinkage parameter, $e = \{1\}^m$ is a vector of ones, $\odot$ is the Hadamard product, repeat(v) defines a new matrix where each row is $v$, and diag(M) is the diagonal of matrix $M$. Division operations involving vectors are performed element-wise. All these operations, including matrix multiplication, have cubic or smaller complexity.

### S3.2.3 Improving the directionality of inferred GRNs

The asymmetric matrix $B$ is combined with the corrected (symmetric) precision matrix $M$ the following way:

$$M_{ij} \leftarrow M_{ij} \frac{B_{ij}}{\max(B_{ij}, B_{ji})} \tag{17}$$

When $B_{ij} > B_{ji}$, the $M_{ij}$ value remains unadjusted, and $M_{ji}$ is reduced by a $B_{ji}/B_{ij}$ ratio, and vice versa. In other words, the $j \rightarrow i$ regulation score is decreased by how low the contribution of gene $j$ is for the prediction of $i$'s expression compared to the contribution of gene $i$ for the prediction of $j$.

The reason for not reporting $B$ as the final score matrix directly, and combining it with $M$ instead, is due to its high empirical error. Indeed, the symmetry of $B$ correlates with the shrinkage parameter $\alpha$, but using a low $\alpha$ increases the error on the estimation of the precision matrix. We therefore propose to solve the dilemma by adjusting $M$ as shown in equation 17. Indeed, the denominator ensures that half of the values in $M$ will remain unchanged, thus preserving the high-quality estimation of partial correlations.

Consequently, PORTIA has two different shrinkage parameters $\alpha_1$ and $\alpha_2$: one for precision matrix estimation, and one for solving the linear regression problems. As proposed in the manuscript, $\alpha_1$ is set to a high value (0.8) in order to start from a well-conditioned shrinkage estimator $\bar{S}$. Conversely, $\alpha_2$ is set to a low value (0.05) to promote the highest asymmetry possible for matrix $B$.

# S4 Computational efficiency

## S4.1 Incomplete precision matrix estimation

Since covariance matrix inversion is PORTIA's bottleneck, we propose not to perform full inversion if a list $L$ of putative TFs is made available. Indeed, the only rows of the precision matrix that need to be estimated are the ones that correspond to genes present in $L$. In such case, Cholesky factorisation is replaced by least-squares estimation of the precision sub-matrix. Let $I \in \{0, 1\}^{m \times m}$ be the identity matrix. The least-squares problem is thus the following:

$$\min_{\Theta} \sum_{i \in L} \sum_{j=1}^{m} \left( I_{ij} - (\bar{S}\Theta)_{ij} \right)^2 \tag{18}$$

The solution to this problem is a rectangular matrix $\Theta$ that is further supplemented with zeroes at the missing indices to make it square.

## S4.2 Cholesky factorisation during end-to-end inference

In etePORTIA, Cholesky factorisation is performed regardless of $L$. Both the computations of the inverse matrix and determinant are the most cost-intensive operations. In consequence, we reused the same Cholesky factorisation $\bar{S} = U^T U$ (where $U$ is an upper-triangular matrix) to compute both the inverse $\Theta = \bar{S}^{-1} = U^{-1}(U^{-1})^T$ and its determinant $\det \Theta = \det U^{-1}(U^{-1})^T = (\det U)^2$.

## S4.3 The Box-Cox transform is only suitable for strictly positive values

Because the Box-Cox transform is undefined at zero, we artificially introduced a positive bias term $\epsilon$. The introduction of such term has the effect of constraining all gene expression data to be strictly positive. However, the value of $\epsilon$ should be chosen carefully,

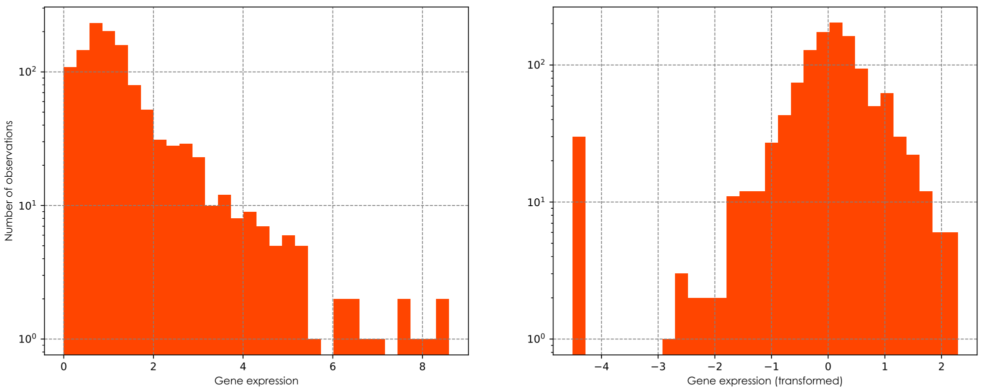


Figure S1: (Left) Histogram of the gene expression values of the 29th gene in the first E. Coli network from the DREAM3 dataset, with logarithmic scale. (Right) Histogram of the same values, after applying a Box-Cox transform ($\epsilon = 10^{-50}$)

as the power transform drastically affects the resulting data. Indeed, genes often follow a zero-inflated distribution, and $\epsilon$ therefore determines how far the zero-associated peak will be located from the mode after transformation.

In Fig S1, the effects of using an arbitrarily-small $\epsilon$ are shown (in log scale). The power transform seems to decrease the skewness, and to isolate the zero-valued observations from the rest. Both effects are desirable, as our method is based on Gaussian modelling, and the difference between knock-out experiments (for a given gene) and other ones needs to be captured. In order to maximize the difference between zero-valued observations and strictly positive values, we chose an arbitrarily-small value of $\epsilon$, namely $10^{-50}$. Let's note that $\epsilon$ should not be smaller, in order to avoid numerical stability issues.

In rare cases, the datasets might have already been pre-processed by their authors, involving standardisation (e.g. MERLIN+P). The resulting data might contain negative values, which cannot be processed by PORTIA. For each gene that contains negative expression values, we added a bias term in such a way that the minimum value becomes 1e-2, in absence of any information about the presence of unexpressed or knocked-out genes.

# S5 Performance assessment

## S5.1 Performance metrics

### S5.1.1 Overall score

The overall score quantifies the significance of inferred networks, based on the AUROC and AUPR scores. It is computed as follows [11, ]:

$$\text{Overall score} = -\frac{1}{2}\log_{10}(\bar{p}_{AUROC}\ \bar{p}_{AUPR}) \tag{19}$$

where $\bar{p}_{AUROC}$ is the mean p-value obtained by comparing the AUROCs of inferred networks against 25,000 randomly generated networks. This p-value is averaged across all

the networks in the dataset (e.g. 3 networks in DREAM5). $\bar{p}_{AUPR}$ is a p-value obtained analogously based on the AUPR score. For DREAM3, DREAM4 and DREAM4MF, the overall score, as well as the AUPR and AUROC scores were computed using the dedicated `dreamtools` Python package [5, ]. For DREAM5, reconstructed networks were evaluated with the help of the MATLAB script available at:
`https://www.synapse.org/#!Synapse:syn2787219`.

Finally, we performed the evaluation on MERLIN+P ourselves, using the `dreamtools` Python package to compute the AUPR and AUROC scores. To compute the p-values, we generated the random networks in such a way that their original sparsity patterns are preserved (gene pairs with no experimental evidence are consistently considered as unknown). We didn't fattened the tail of the corresponding distribution using a stretched exponential, but smoothed it by kernel density estimation. We used an exponential kernel with a bandwidth parameter equal to half the standard deviation.

#### S5.1.2 Normalised discounted cumulative gain

Given the number $k$ of known interactions in the goldstandard network, an inferred GRN matrix $M$ and the indices $I = \{(i_1, j_1), (i_2, j_2), \dots\}$ of $M$'s elements sorted by decreasing value, the discounted cumulative gain is defined as:

$$DCG_k(M) = \sum_{b=1, I_b=(i,j)}^{k} \frac{1}{\log_2(1+b)} R_{ij} \tag{20}$$

where $R_{ij}$ is the relevance of the regulatory relation $i \to j$, given the underlying causal structure. Each regulatory relationship is weighted according to its ranking in the inferred GRN.

We define 5 categories of causal structures, listed by decreasing degree of relevance:

- *True positives*: the first gene directly regulates the second one.
- *Chains*: the first gene indirectly regulates the second one.
- *D-connected genes* (that are not part of a regulatory chain): the two genes are d-connected, allowing their expression to be correlated. Two genes A and B are d-connected if there is a TF C that regulates both of them. Genes in this category can either be located downstream of a *fork* or at the ends of a *reversed chain*.
- *D-separated genes*: the two genes are not d-connected, there is no confounder gene that indirectly regulates both genes. This category contains *colliders* and more generally *undirected relations* that do not fall in the previous categories (e.g. $A \leftarrow B \to C \leftarrow D$).
- *Spurious correlations*: given all known interactions from the goldstandard, there is no (undirected) path linking the two genes. The two genes are, to the best of current knowledge, completely unrelated.

When looking for the causal structure that could most likely explain the predicted gene pair, a specific order should be followed:

- *True positives* should be checked first. The (A, B) link has been inferred, and there is evidence for the regulatory relationship $A \to B$ in the goldstandard network.

- *Chains* should be checked next. The (A, B) link has been inferred, and the relationship $A \rightarrow \ldots \rightarrow B$ exists.
- *Reversed chains* should be checked before *forks*, as they can be seen as a particular case of *fork*. We refer to *reversed chains* as regulatory chains containing two genes for which a false positive corresponds to a direct regulatory relationship inferred in the wrong direction. The (A, B) link has been inferred, and the relationship $A \leftarrow \ldots \leftarrow B$ exists.
- *Forks*: when the two reported genes are regulated by the same TF. The (A, B) link has been inferred, and the structure $A \leftarrow \ldots \leftarrow C \rightarrow \ldots \rightarrow B$ exists.
- *Colliders* should be checked before *undirected relationships*, as all structures (except spurious correlations) are a specific case of *undirected relationships*. The (A, B) link has been inferred, and the structure $A \rightarrow \ldots \rightarrow C \leftarrow \ldots \leftarrow B$ exists.
- *Undirected relationships*: any pair of genes that are connected, regardless of edge directionality. The (A, B) link has been inferred, and the undirected relationship $A - \ldots - B$ exists.
- Spurious correlations: the (A, B) link has been inferred, but there is no evidence for a $A - \ldots - B$ relationship in the goldstandard network.

We assigned 0 to the *spurious correlations* category and arbitrarily decided to assign the other weights exponentially. More specifically, the 5 categories (true positives, chains, d-connected, d-separated and spurious correlations) are assigned the scores 4, 2, 1, $\frac{1}{2}$ and 0, respectively.

The ideal discounted cumulative gain (IDCG) is defined as the DCG upper bound, given by:

$$IDCG_k = \sum_{b=1}^{k} \frac{4}{\log_2(1+b)} \tag{21}$$

The normalized discounted cumulative factor is then formulated as $NDCG_k(M) = DCG_k(M)/IDCG_k$.

### S5.1.3 Matrix symmetry

We used matrix symmetry as a proxy for assessing the ability of an algorithm to learn directional information from gene expression data. Matrix symmetry is computed on the raw predictions (continuous values), and we define it as:

$$s = \frac{\|M_{sym}\|_F - \|M_{anti}\|_F}{\|M_{sym}\|_F + \|M_{anti}\|_F} \tag{22}$$

where $\|\cdot\|_F$ is the Frobenius norm, $M_{sym} = \frac{1}{2}(A + A^T)$ is the symmetric part of matrix $M$ and $M_{anti} = \frac{1}{2}(A - A^T)$ its antisymmetric part. By construction, $M = M_{sym} + M_{anti}$. It must be noted that this metric ranges between -1 and 1, where 1 corresponds to $M$ being perfectly symmetric, and -1 corresponds to $M$ being perfectly antisymmetric.

Table S4: Proportions of false positives made on DREAM3, categorised by the local causal structure in which they occurred.

| Structure | Illustration | Network | ARACNe-AP | GENIE3 | PLSNET | TIGRESS | ENNET | PORTIA | etePORTIA |
|---|---|---|---|---|---|---|---|---|---|
| | | Net1 | 7 | 3 | 6 | 16 | 58 | 89 | **90** |
| | | Net2 | 8 | 2 | 8 | 13 | 68 | **94** | **94** |
| "True positive" | - | Net3 | 13 | 3 | 11 | 17 | 63 | **88** | **88** |
| | | Net4 | 25 | 10 | 32 | 40 | 122 | **206** | 204 |
| | | Net5 | 40 | 40 | 58 | 50 | 141 | **234** | **234** |
| | | Net1 | 0 | 0 | 0 | 0 | 0 | 23 | **24** |
| | | Net2 | 1 | 0 | 0 | 1 | 5 | **20** | **20** |
| "Chain" | 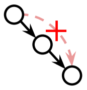 | Net3 | 8 | 10 | 11 | 9 | 20 | **45** | **45** |
| | | Net4 | 75 | 90 | 100 | 82 | 88 | 164 | **166** |
| | | Net5 | 98 | 103 | 85 | 107 | 131 | **210** | **210** |
| | | Net1 | 49 | 51 | **61** | 51 | 36 | 2 | 2 |
| | | Net2 | 2 | 29 | **32** | 29 | 13 | 0 | 0 |
| "Fork" | 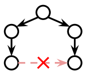 | Net3 | 10 | 48 | **54** | 51 | 32 | 9 | 9 |
| | | Net4 | 78 | **119** | 113 | **119** | 75 | 6 | 6 |
| | | Net5 | 118 | **147** | 139 | 137 | 91 | 22 | 21 |
| | | Net1 | **4** | **4** | 0 | 0 | 1 | 0 | 0 |
| | | Net2 | 0 | **2** | 0 | 0 | 0 | 0 | 0 |
| "Collider" | 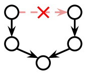 | Net3 | **29** | 12 | 11 | 13 | 6 | 1 | 1 |
| | | Net4 | **111** | 57 | 63 | 57 | 37 | 5 | 5 |
| | | Net5 | **116** | 84 | 89 | 95 | 73 | 45 | 46 |
| | | Net1 | 7 | 3 | 6 | **9** | 4 | 0 | 0 |
| | | Net2 | **9** | 8 | 4 | 7 | 2 | 0 | 0 |
| "Chain (reversed)" | 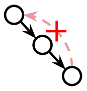 | Net3 | **21** | 19 | 18 | 16 | 10 | 5 | 5 |
| | | Net4 | 100 | **113** | 80 | 89 | 66 | 7 | 7 |
| | | Net5 | 137 | 156 | **163** | 134 | 97 | 29 | 27 |
| | | Net1 | 28 | **29** | 26 | 25 | 14 | 11 | 9 |
| | | Net2 | **49** | 40 | 36 | 39 | 15 | 2 | 2 |
| "Undirected" | 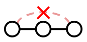 | Net3 | **43** | 35 | 34 | 33 | 23 | 9 | 9 |
| | | Net4 | 0 | 0 | **1** | **1** | 0 | 0 | 0 |
| | | Net5 | **21** | 13 | 10 | 11 | 10 | 2 | 3 |
| | | Net1 | 30 | **35** | 26 | 24 | 12 | 0 | 0 |
| | | Net2 | **50** | 38 | 39 | 30 | 16 | 3 | 3 |
| "Spurious correlation" | 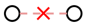 | Net3 | **42** | 39 | 27 | 27 | 12 | 9 | 9 |
| | | Net4 | 0 | 0 | 0 | **1** | **1** | **1** | **1** |
| | | Net5 | **21** | 8 | 7 | 17 | 8 | 9 | 10 |

Table S5: Proportions of false positives made on DREAM4, categorised by the local causal structure in which they occurred.

| Structure | Illustration | Network | ARACNe-AP | GENIE3 | PLSNET | TIGRESS | ENNET | PORTIA | etePORTIA |
|---|---|---|---|---|---|---|---|---|---|
| "True positive" | - | Net1 | 18 | 29 | 16 | 24 | 81 | **103** | **103** |
| | | Net2 | 36 | 44 | 19 | 31 | 99 | **131** | **131** |
| | | Net3 | 38 | 50 | 29 | 52 | **89** | 87 | 85 |
| | | Net4 | 30 | 39 | 25 | 33 | 89 | **106** | 103 |
| | | Net5 | 35 | 44 | 25 | 42 | **69** | 64 | 62 |
| "Chain" | 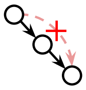 | Net1 | 7 | 2 | 1 | 4 | 18 | **62** | 61 |
| | | Net2 | 16 | 18 | 9 | 17 | 25 | **57** | 56 |
| | | Net3 | 63 | 38 | 27 | 30 | 53 | **83** | 82 |
| | | Net4 | 42 | 32 | 27 | 35 | 40 | 101 | **102** |
| | | Net5 | 18 | 19 | 16 | 12 | 41 | 116 | **117** |
| "Fork" | 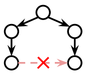 | Net1 | **125** | 98 | 105 | 99 | 50 | 0 | 0 |
| | | Net2 | **131** | 103 | 130 | 114 | 68 | 18 | 16 |
| | | Net3 | 36 | 52 | **76** | 62 | 24 | 5 | 7 |
| | | Net4 | 83 | 77 | **106** | 80 | 46 | 1 | 1 |
| | | Net5 | 84 | 82 | **101** | 88 | 53 | 3 | 2 |
| "Collider" | 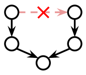 | Net1 | 0 | 2 | 2 | **3** | 2 | 1 | 1 |
| | | Net2 | 6 | 6 | 6 | 3 | 5 | 11 | **12** |
| | | Net3 | 0 | 0 | 4 | 2 | 1 | **10** | 9 |
| | | Net4 | 2 | 7 | 3 | **9** | 6 | 2 | 2 |
| | | Net5 | **6** | 1 | 1 | 3 | 1 | 1 | 0 |
| "Chain (reversed)" | 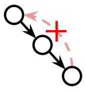 | Net1 | 22 | **29** | 15 | 22 | 13 | 1 | 2 |
| | | Net2 | **47** | 38 | 27 | 36 | 18 | 4 | 3 |
| | | Net3 | **58** | 50 | 35 | 38 | 22 | 2 | 4 |
| | | Net4 | **54** | 45 | 37 | 40 | 21 | 0 | 0 |
| | | Net5 | **48** | 27 | 19 | 27 | 14 | 1 | 1 |
| "Undirected" | 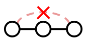 | Net1 | 2 | 7 | **11** | 10 | 4 | 3 | 3 |
| | | Net2 | 7 | 14 | **40** | 25 | 14 | 11 | 14 |
| | | Net3 | 0 | 2 | **13** | 5 | 3 | 7 | 7 |
| | | Net4 | 0 | 6 | 5 | **7** | 5 | 0 | 2 |
| | | Net5 | 1 | 8 | **16** | 12 | 6 | 3 | 5 |
| "Spurious correlation" | 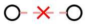 | Net1 | 2 | 9 | **26** | 14 | 8 | 6 | 6 |
| | | Net2 | 6 | **26** | 18 | 23 | 20 | 17 | 17 |
| | | Net3 | 0 | 3 | **11** | 6 | 3 | 1 | 1 |
| | | Net4 | 0 | 5 | **8** | 7 | 4 | 1 | 1 |
| | | Net5 | 1 | 12 | **15** | 9 | 9 | 5 | 6 |

Table S6: Proportions of false positives made on DREAM4MF, categorised by the local causal structure in which they occurred.

| Structure | Illustration | Network | ARACNe-AP | GENIE3 | PLSNET | TIGRESS | ENNET | PORTIA | etePORTIA |
|---|---|---|---|---|---|---|---|---|---|
| "True positive" | - | Net1 | 38 | 38 | 31 | 38 | **43** | 32 | 35 |
| | | Net2 | 38 | 60 | **92** | 64 | 86 | 60 | 61 |
| | | Net3 | 56 | 64 | 59 | 65 | **73** | 58 | 60 |
| | | Net4 | 54 | 63 | 59 | 62 | **71** | 67 | 67 |
| | | Net5 | 51 | 50 | 51 | **60** | **60** | 49 | 48 |
| "Chain" | 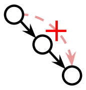 | Net1 | 3 | **4** | **4** | **4** | **4** | **4** | **4** |
| | | Net2 | 14 | 11 | 14 | 9 | **16** | 9 | 9 |
| | | Net3 | 47 | 48 | **62** | 44 | 58 | 45 | 45 |
| | | Net4 | 39 | 38 | 55 | 37 | **71** | 37 | 41 |
| | | Net5 | 19 | 26 | 10 | 17 | **45** | 9 | 10 |
| "Fork" | 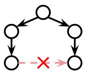 | Net1 | 68 | **83** | 61 | 79 | 57 | 61 | 62 |
| | | Net2 | 104 | 100 | 90 | 98 | 75 | 105 | **107** |
| | | Net3 | 31 | 25 | 29 | 35 | 24 | **45** | **45** |
| | | Net4 | **61** | 57 | 42 | 57 | 30 | 54 | 52 |
| | | Net5 | 55 | 65 | 64 | 59 | 44 | 71 | **72** |
| "Collider" | 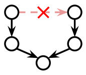 | Net1 | **4** | 1 | 2 | 0 | 2 | 3 | 3 |
| | | Net2 | **19** | 0 | 6 | 2 | 7 | 4 | 4 |
| | | Net3 | 2 | 1 | 5 | 0 | 2 | **6** | 4 |
| | | Net4 | 0 | 0 | **10** | 1 | 4 | 7 | 7 |
| | | Net5 | 4 | 4 | **6** | 1 | 5 | 4 | 3 |
| "Chain (reversed)" | 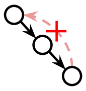 | Net1 | 35 | **37** | 20 | 35 | 23 | 30 | 28 |
| | | Net2 | 46 | **62** | 26 | 52 | 32 | 29 | 32 |
| | | Net3 | **59** | 57 | 36 | 48 | 38 | 37 | 37 |
| | | Net4 | **57** | 53 | 36 | 53 | 32 | 42 | 40 |
| | | Net5 | **58** | 48 | 26 | 46 | 33 | 22 | 22 |
| "Undirected" | 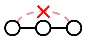 | Net1 | 11 | 5 | 14 | 10 | 7 | 19 | **20** |
| | | Net2 | 14 | 7 | 9 | 12 | 20 | **21** | 18 |
| | | Net3 | 0 | 0 | **2** | **2** | 0 | **2** | **2** |
| | | Net4 | 0 | 0 | **3** | 0 | 1 | **3** | **3** |
| | | Net5 | 3 | 0 | **24** | 5 | 6 | **24** | 22 |
| "Spurious correlation" | 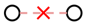 | Net1 | 17 | 8 | **44** | 10 | 40 | 27 | 24 |
| | | Net2 | 14 | 9 | 12 | 12 | 13 | **21** | 18 |
| | | Net3 | 0 | 0 | **2** | 1 | 0 | **2** | **2** |
| | | Net4 | 0 | 0 | **6** | 1 | 2 | 1 | 1 |
| | | Net5 | 3 | 0 | 12 | 5 | 0 | 14 | **16** |

Table S7: Proportions of false positives made on DREAM5, categorised by the local causal structure in which they occurred.

| Structure | Illustration | Network | ARACNe-AP | GENIE3 | PLSNET | TIGRESS | ENNET | PORTIA | etePORTIA |
|---|---|---|---|---|---|---|---|---|---|
| "True positive" | - | Net1 | 1039 | 1352 | 1267 | 1341 | **1792** | 1699 | 1712 |
| | | Net3 | 75 | **163** | 113 | 121 | 69 | 145 | 148 |
| | | Net4 | 13 | 29 | 31 | 32 | 12 | **90** | **90** |
| "Chain" | 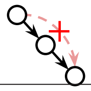 | Net1 | 207 | 303 | **737** | 196 | 482 | 445 | 455 |
| | | Net3 | 7 | 11 | **29** | 9 | 6 | 8 | 8 |
| | | Net4 | 22 | 19 | 16 | 31 | 24 | **42** | **42** |
| "Fork" | 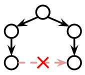 | Net1 | **2253** | 1645 | 1578 | 1466 | 1019 | 996 | 1000 |
| | | Net3 | 60 | 123 | 253 | 151 | 88 | **310** | 308 |
| | | Net4 | 340 | 400 | 291 | 369 | 319 | 568 | **572** |
| "Collider" | 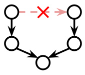 | Net1 | 68 | 55 | 35 | 47 | 42 | **120** | 115 |
| | | Net3 | 4 | **6** | 3 | 4 | 4 | 4 | 4 |
| | | Net4 | 12 | 14 | 9 | 9 | 5 | **20** | 19 |
| "Chain (reversed)" | 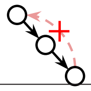 | Net1 | **91** | 83 | 57 | 65 | 49 | 45 | 45 |
| | | Net3 | 2 | 4 | **7** | 3 | 2 | 5 | 5 |
| | | Net4 | 7 | 6 | 4 | 5 | 1 | **12** | **12** |
| "Undirected" | 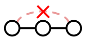 | Net1 | 23 | 61 | 8 | 58 | 34 | **81** | 78 |
| | | Net3 | 2 | 4 | 7 | **9** | 5 | 8 | 8 |
| | | Net4 | **19** | 15 | 13 | **19** | 11 | 16 | 16 |
| "Spurious correlation" | 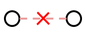 | Net1 | 331 | 513 | 330 | **839** | 594 | 626 | 607 |
| | | Net3 | **1916** | 1755 | 1654 | 1769 | 1892 | 1586 | 1585 |
| | | Net4 | 3527 | 3457 | **3576** | 3475 | 3568 | 3192 | 3189 |

Table S8: Proportions of false positives made on the MERLIN+P datasets, categorised by the local causal structure in which they occurred.

| Structure | Illustration | Network | ARACNe-AP | GENIE3 | PLSNET | TIGRESS | ENNET | PORTIA | etePORTIA |
|---|---|---|---|---|---|---|---|---|---|
| | | niu (Cusanovich) | 21 | 28 | 27 | **59** | 12 | 42 | 43 |
| | | Geuvadis (Cusanovich) | 40 | 40 | 11 | 57 | **60** | 31 | 29 |
| | | NatVar (MacIsaac2) | 54 | 12 | 48 | 69 | 65 | 180 | **182** |
| | | NatVar (YEASTRACT_Count3) | 76 | 17 | 32 | 26 | 71 | 214 | **216** |
| | | NatVar (YEASTRACT_Type2) | 80 | 37 | 40 | 18 | 79 | 229 | **231** |
| "True positive" | - | KO (MacIsaac2) | 29 | 27 | 23 | 41 | 16 | **48** | 45 |
| | | KO (YEASTRACT_Count3) | 37 | 20 | 3 | 30 | 12 | 59 | **60** |
| | | KO (YEASTRACT_Type2) | 35 | 28 | 7 | **68** | 19 | 55 | 54 |
| | | Stress (MacIsaac2) | 28 | 30 | 30 | 22 | 35 | **59** | **59** |
| | | Stress (YEASTRACT_Count3) | 24 | 22 | 20 | 21 | 28 | **70** | **70** |
| | | Stress (YEASTRACT_Type2) | 47 | 27 | 45 | 23 | 19 | **73** | 72 |
| | | niu (Cusanovich) | 20 | 29 | 14 | **136** | 3 | 76 | 78 |
| | | Geuvadis (Cusanovich) | 105 | 8 | 187 | 101 | **210** | 78 | 80 |
| | | NatVar (MacIsaac2) | 61 | 10 | 70 | **146** | 69 | 116 | 114 |
| | | NatVar (YEASTRACT_Count3) | 38 | 4 | 23 | 40 | 6 | 54 | **56** |
| | | NatVar (YEASTRACT_Type2) | 84 | 66 | **193** | 59 | 29 | 51 | 51 |
| "Chain" | 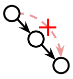 | KO (MacIsaac2) | **187** | 86 | 36 | 155 | 101 | 125 | 126 |
| | | KO (YEASTRACT_Count3) | 41 | **54** | 2 | 35 | 7 | 37 | 40 |
| | | KO (YEASTRACT_Type2) | 72 | 17 | 8 | 83 | 26 | 81 | **86** |
| | | Stress (MacIsaac2) | 116 | **118** | 48 | 95 | 24 | 112 | 112 |
| | | Stress (YEASTRACT_Count3) | 11 | **137** | 41 | 25 | 2 | 36 | 36 |
| | | Stress (YEASTRACT_Type2) | 58 | **94** | 6 | 60 | 3 | 77 | 75 |
| | | niu (Cusanovich) | 5837 | 6053 | **6113** | 5723 | 5998 | 5791 | 5788 |
| | | Geuvadis (Cusanovich) | 5678 | **5995** | 5722 | 5625 | 5706 | 5843 | 5843 |
| | | NatVar (MacIsaac2) | 1274 | 1340 | 1392 | 1378 | **1768** | 1702 | 1707 |
| | | NatVar (YEASTRACT_Count3) | 984 | 1203 | 1411 | 1217 | 1429 | 1606 | **1612** |
| | | NatVar (YEASTRACT_Type2) | 1932 | 2104 | 1222 | 2248 | **2899** | 2635 | 2631 |
| "Fork" | 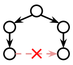 | KO (MacIsaac2) | 1323 | 1541 | **1888** | 1431 | 1670 | 1468 | 1476 |
| | | KO (YEASTRACT_Count3) | 1266 | 1016 | **1915** | 1179 | 1698 | 1433 | 1436 |
| | | KO (YEASTRACT_Type2) | 1821 | 2621 | **2768** | 1897 | 2081 | 2226 | 2225 |
| | | Stress (MacIsaac2) | 1637 | 1343 | 1816 | 1356 | **1924** | 1574 | 1576 |
| | | Stress (YEASTRACT_Count3) | 1512 | 1260 | 1278 | 1171 | **1965** | 1713 | 1716 |
| | | Stress (YEASTRACT_Type2) | 2793 | 2760 | 2807 | 2046 | **2952** | 2576 | 2576 |
| | | niu (Cusanovich) | 0 | 0 | 0 | **2** | 0 | 0 | 0 |
| | | Geuvadis (Cusanovich) | 0 | 0 | **1** | 0 | 0 | 0 | 0 |
| | | NatVar (MacIsaac2) | 10 | 8 | 17 | 32 | 6 | **41** | **41** |
| | | NatVar (YEASTRACT_Count3) | 4 | 14 | 27 | 21 | 11 | **31** | 28 |
| | | NatVar (YEASTRACT_Type2) | 10 | 20 | **46** | 22 | 13 | 30 | 31 |
| "Collider" | 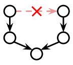 | KO (MacIsaac2) | **44** | 23 | 10 | 34 | 15 | 33 | 31 |
| | | KO (YEASTRACT_Count3) | 22 | 12 | 5 | **24** | 1 | 20 | 20 |
| | | KO (YEASTRACT_Type2) | 28 | 12 | 17 | 9 | 7 | 33 | **34** |
| | | Stress (MacIsaac2) | 12 | 13 | 14 | **39** | 11 | 28 | 29 |
| | | Stress (YEASTRACT_Count3) | 0 | 19 | 22 | **27** | 2 | 21 | 22 |
| | | Stress (YEASTRACT_Type2) | 2 | 8 | **25** | 15 | 2 | 22 | 20 |
| | | niu (Cusanovich) | 10 | **23** | 17 | 21 | 16 | **23** | **23** |
| | | Geuvadis (Cusanovich) | **28** | 18 | 16 | 17 | 20 | 22 | 23 |
| | | NatVar (MacIsaac2) | 4 | 10 | **35** | 17 | 13 | 29 | 29 |
| | | NatVar (YEASTRACT_Count3) | 6 | 2 | 5 | 7 | 8 | **14** | **14** |
| | | NatVar (YEASTRACT_Type2) | 5 | 9 | 9 | 11 | 10 | 11 | **12** |
| "Chain (reversed)" | 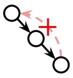 | KO (MacIsaac2) | 20 | 30 | **45** | 22 | 17 | 21 | 21 |
| | | KO (YEASTRACT_Count3) | 7 | 4 | **10** | 1 | 5 | 5 | 5 |
| | | KO (YEASTRACT_Type2) | 9 | 9 | **18** | 8 | 6 | 11 | 11 |
| | | Stress (MacIsaac2) | 17 | 23 | **57** | 12 | 23 | 19 | 19 |
| | | Stress (YEASTRACT_Count3) | 1 | 10 | 11 | 4 | 8 | **12** | **12** |
| | | Stress (YEASTRACT_Type2) | 10 | 10 | **17** | 12 | 12 | 14 | 15 |
| | | niu (Cusanovich) | **495** | 250 | 212 | 442 | 354 | 451 | 451 |
| | | Geuvadis (Cusanovich) | 532 | 322 | 446 | **583** | 387 | 409 | 408 |
| | | NatVar (MacIsaac2) | **2368** | 2339 | 2211 | 2117 | 1849 | 1703 | 1698 |
| | | NatVar (YEASTRACT_Count3) | **2693** | 2561 | 2303 | 2490 | 2276 | 1882 | 1875 |
| | | NatVar (YEASTRACT_Type2) | 2075 | 1957 | **2678** | 1825 | 1166 | 1223 | 1223 |
| "Undirected" | 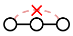 | KO (MacIsaac2) | **2171** | 2046 | 1576 | 2026 | 1954 | 2052 | 2048 |
| | | KO (YEASTRACT_Count3) | 2413 | **2680** | 1851 | 2517 | 2063 | 2232 | 2225 |
| | | KO (YEASTRACT_Type2) | **2178** | 1483 | 1354 | 2094 | 1972 | 1729 | 1725 |
| | | Stress (MacIsaac2) | 1917 | 2060 | 1633 | **2170** | 1711 | 1921 | 1916 |
| | | Stress (YEASTRACT_Count3) | 2293 | 2393 | 2469 | **2593** | 1836 | 1989 | 1985 |
| | | Stress (YEASTRACT_Type2) | 1337 | 1345 | 1342 | **2076** | 1262 | 1452 | 1457 |
| | | niu (Cusanovich) | **0** | **0** | **0** | **0** | **0** | **0** | **0** |
| | | Geuvadis (Cusanovich) | **0** | **0** | **0** | **0** | **0** | **0** | **0** |
| | | NatVar (MacIsaac2) | 15 | **67** | 13 | 27 | 16 | 15 | 15 |
| | | NatVar (YEASTRACT_Count3) | **0** | **0** | **0** | **0** | **0** | **0** | **0** |
| | | NatVar (YEASTRACT_Type2) | 15 | 8 | 13 | 18 | 5 | **22** | **22** |
| "Spurious correlation" | 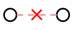 | KO (MacIsaac2) | 21 | 42 | **217** | 86 | 22 | 48 | 48 |
| | | KO (YEASTRACT_Count3) | **0** | **0** | **0** | **0** | **0** | **0** | **0** |
| | | KO (YEASTRACT_Type2) | 40 | 13 | 11 | 24 | **72** | 48 | 48 |
| | | Stress (MacIsaac2) | 29 | **169** | 158 | 62 | 28 | 43 | 45 |
| | | Stress (YEASTRACT_Count3) | **0** | **0** | **0** | **0** | **0** | **0** | **0** |
| | | Stress (YEASTRACT_Type2) | 10 | 13 | 15 | 25 | 7 | **43** | 42 |

# S6 Results

## S6.1 Performance on MERLIN+P datasets

Table S9: AUPR and AUROC scores of different GRN methods, with corresponding p-values computed on 25000 randomly-generated networks on the LCL dataset.

| Method | LCL (Niu) | | | | LCL (Geuvadis) | | | |
|---|---|---|---|---|---|---|---|---|
| | AUPR | p-value | AUROC | p-value | AUPR | p-value | AUROC | p-value |
| ARACNe-AP | 0.137 | 4.16e-01 | 0.503 | 3.04e-01 | 0.134 | 8.14e-01 | 0.493 | 8.87e-01 |
| GENIE3 | 0.125 | 1.00e+00 | 0.482 | 9.98e-01 | 0.137 | 3.84e-01 | 0.501 | 4.29e-01 |
| PLSNET | 0.130 | 9.84e-01 | 0.484 | 9.95e-01 | 0.118 | 1.00e+00 | 0.468 | 1.00e+00 |
| TIGRESS | 0.138 | 3.13e-01 | 0.500 | 4.98e-01 | **0.150** | **4.05e-05** | **0.520** | **1.18e-03** |
| ENNET | 0.128 | 9.98e-01 | 0.491 | 9.37e-01 | 0.128 | 9.97e-01 | 0.483 | 9.96e-01 |
| PORTIA | 0.140 | 9.63e-02 | 0.502 | 3.73e-01 | 0.141 | 5.46e-02 | 0.502 | 3.66e-01 |
| etePORTIA | **0.140** | **8.55e-02** | **0.509** | **5.88e-02** | 0.140 | 9.76e-02 | 0.505 | 1.94e-01 |

Table S10: AUPR and AUROC scores of different GRN methods, with corresponding p-values computed on 25000 randomly-generated networks on the NatVar dataset.

| Method | MacIsaac2 | | | | YEASTRACT Count3 | | | | YEASTRACT Type2 | | | |
|---|---|---|---|---|---|---|---|---|---|---|---|---|
| | AUPR | p-value | AUROC | p-value | AUPR | p-value | AUROC | p-value | AUPR | p-value | AUROC | p-value |
| ARACNe-AP | 0.030 | 1.21e-09 | 0.552 | 1.16e-03 | 0.037 | 1.28e-17 | 0.587 | 1.06e-05 | 0.035 | 1.08e-14 | 0.595 | 2.13e-06 |
| GENIE3 | 0.016 | 8.88e-01 | 0.495 | 6.20e-01 | 0.014 | 8.71e-01 | 0.467 | 9.77e-01 | 0.016 | 5.99e-01 | 0.480 | 8.97e-01 |
| PLSNET | 0.045 | 0.00e+00 | 0.574 | 4.02e-05 | 0.020 | 4.49e-04 | 0.508 | 3.02e-01 | 0.032 | 1.40e-12 | 0.486 | 8.14e-01 |
| TIGRESS | 0.032 | 1.86e-11 | 0.530 | 2.76e-02 | 0.015 | 6.95e-01 | 0.507 | 3.45e-01 | 0.013 | 9.89e-01 | 0.456 | 9.95e-01 |
| ENNET | 0.046 | 0.00e+00 | 0.582 | 1.22e-05 | 0.029 | 7.06e-11 | 0.554 | 1.34e-03 | 0.037 | 5.62e-16 | 0.571 | 8.26e-05 |
| PORTIA | **0.095** | **0.00e+00** | 0.651 | 2.59e-10 | **0.119** | **0.00e+00** | 0.660 | 2.48e-10 | 0.118 | 0.00e+00 | 0.661 | 1.08e-10 |
| etePORTIA | **0.095** | **0.00e+00** | **0.652** | **2.23e-10** | **0.119** | **0.00e+00** | **0.661** | **2.05e-10** | **0.119** | **0.00e+00** | **0.665** | **5.58e-11** |

Table S11: AUPR and AUROC scores of different GRN methods, with corresponding p-values computed on 25000 randomly-generated networks on the KO dataset.

| Method | MacIsaac2 | | | | YEASTRACT Count3 | | | | YEASTRACT Type2 | | | |
|---|---|---|---|---|---|---|---|---|---|---|---|---|
| | AUPR | p-value | AUROC | p-value | AUPR | p-value | AUROC | p-value | AUPR | p-value | AUROC | p-value |
| ARACNe-AP | 0.017 | 5.06e-01 | 0.498 | 5.52e-01 | 0.021 | 4.88e-04 | 0.543 | 6.61e-03 | 0.019 | 5.66e-02 | 0.523 | 7.10e-02 |
| GENIE3 | 0.019 | 1.80e-01 | 0.528 | 3.78e-02 | 0.014 | 8.62e-01 | 0.493 | 6.73e-01 | 0.016 | 6.15e-01 | 0.497 | 5.78e-01 |
| PLSNET | 0.018 | 3.10e-01 | 0.502 | 4.51e-01 | 0.014 | 8.62e-01 | 0.486 | 8.11e-01 | 0.013 | 9.96e-01 | 0.476 | 9.32e-01 |
| TIGRESS | 0.020 | 1.74e-02 | 0.514 | 1.88e-01 | 0.015 | 5.24e-01 | 0.499 | 5.19e-01 | 0.025 | 4.71e-06 | 0.548 | 2.42e-03 |
| ENNET | 0.026 | 2.22e-06 | 0.525 | 5.47e-02 | 0.024 | 1.88e-06 | 0.519 | 1.26e-01 | 0.021 | 2.16e-03 | 0.518 | 1.29e-01 |
| PORTIA | **0.026** | **1.71e-06** | 0.539 | 7.46e-03 | **0.030** | **2.37e-11** | **0.563** | **3.29e-04** | **0.030** | **3.56e-10** | **0.552** | **1.26e-03** |
| etePORTIA | 0.025 | 3.74e-06 | **0.542** | **5.58e-03** | 0.030 | 4.34e-11 | 0.563 | 3.57e-04 | 0.029 | 7.44e-10 | 0.550 | 1.75e-03 |

Table S12: AUPR and AUROC scores of different GRN methods, with corresponding p-values computed on 25000 randomly-generated networks on the StressResp dataset.

| Method | MacIsaac2 | | | | YEASTRACT Count3 | | | | YEASTRACT Type2 | | | |
|---|---|---|---|---|---|---|---|---|---|---|---|---|
| | AUPR | p-value | AUROC | p-value | AUPR | p-value | AUROC | p-value | AUPR | p-value | AUROC | p-value |
| ARACNe-AP | 0.021 | 4.74e-03 | 0.534 | 1.63e-02 | 0.020 | 5.24e-04 | 0.549 | 2.55e-03 | 0.024 | 1.63e-05 | **0.562** | **3.22e-04** |
| GENIE3 | 0.019 | 6.77e-02 | 0.524 | 6.28e-02 | 0.014 | 8.56e-01 | 0.498 | 5.37e-01 | 0.015 | 8.11e-01 | 0.483 | 8.59e-01 |
| PLSNET | 0.022 | 2.05e-03 | 0.519 | 1.09e-01 | 0.014 | 7.10e-01 | 0.513 | 1.98e-01 | 0.021 | 6.41e-04 | 0.509 | 2.85e-01 |
| TIGRESS | 0.016 | 8.53e-01 | 0.496 | 6.15e-01 | 0.010 | 1.00e+00 | 0.478 | 9.04e-01 | 0.019 | 1.87e-02 | 0.517 | 1.47e-01 |
| ENNET | 0.027 | 1.26e-06 | **0.552** | **1.06e-03** | 0.028 | 1.14e-10 | 0.526 | 5.53e-02 | 0.028 | 4.79e-09 | 0.529 | 4.09e-02 |
| PORTIA | 0.029 | 7.42e-09 | 0.543 | 4.61e-03 | 0.032 | 3.02e-13 | 0.580 | 2.75e-05 | 0.032 | 2.59e-12 | 0.554 | 1.11e-03 |
| etePORTIA | **0.030** | **4.44e-09** | 0.543 | 4.25e-03 | **0.032** | **2.49e-13** | **0.581** | **2.55e-05** | **0.032** | **1.41e-12** | 0.552 | 1.48e-03 |

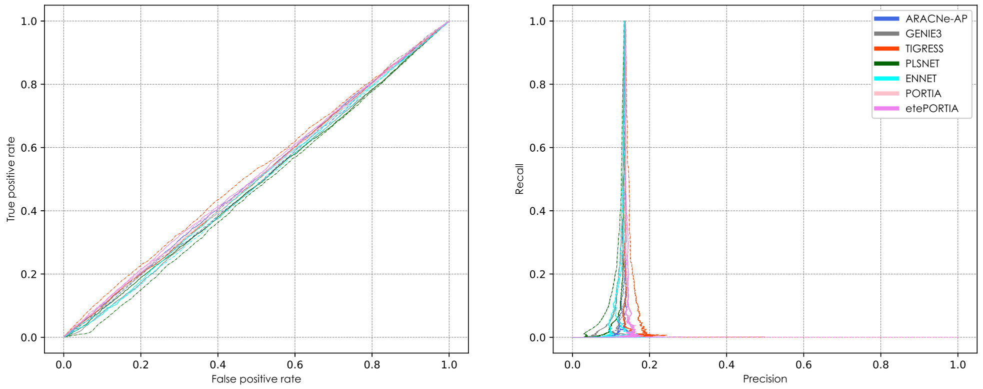


Figure S2: (Left) Receiver operating characteristic (Right) and Precision-recall curves of different GRN inferences methods on the LCL datasets. Dense and dashed lines correspond to the expression datasets from Niu and Geuvadis, respectively.

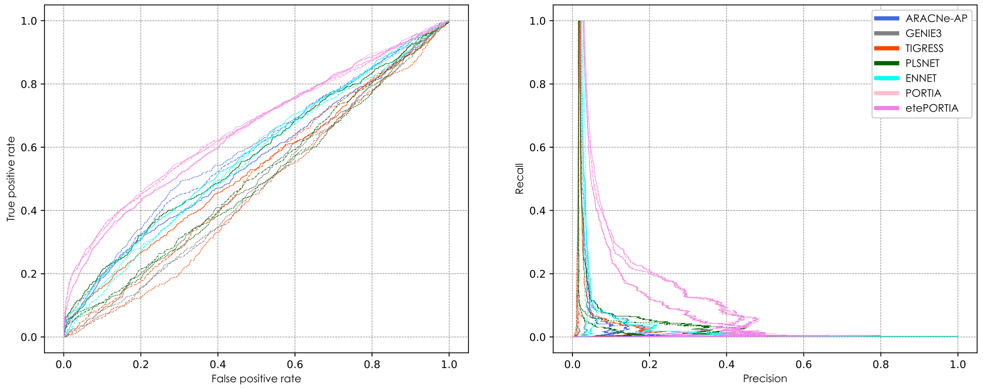


Figure S3: (Left) Receiver operating characteristic (Right) and Precision-recall curves of different GRN inferences methods on the NatVar expression data. Dense, dot-dashed and dashed lines correspond to the MacIsaac2, YEASTRACT Count3 and YEASTRACT Type2 goldstandard networks, respectively.

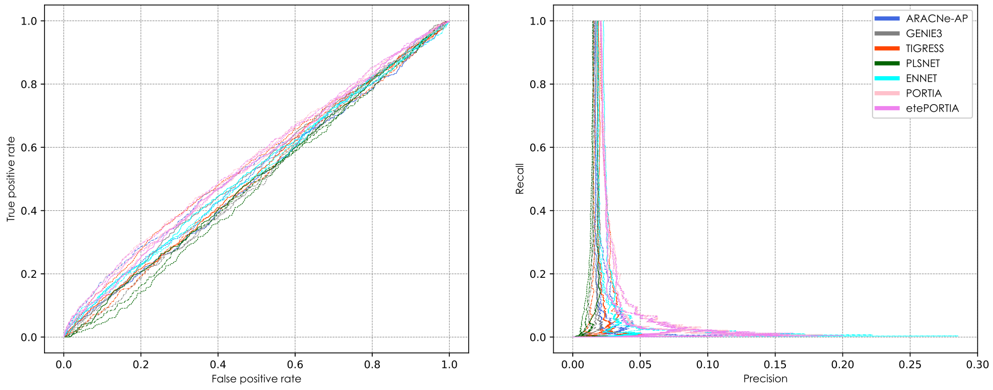


Figure S4: (Left) Receiver operating characteristic (Right) and Precision-recall curves of different GRN inferences methods on the KO expression data. Dense, dot-dashed and dashed lines correspond to the MacIsaac2, YEASTRACT Count3 and YEASTRACT Type2 goldstandard networks, respectively.

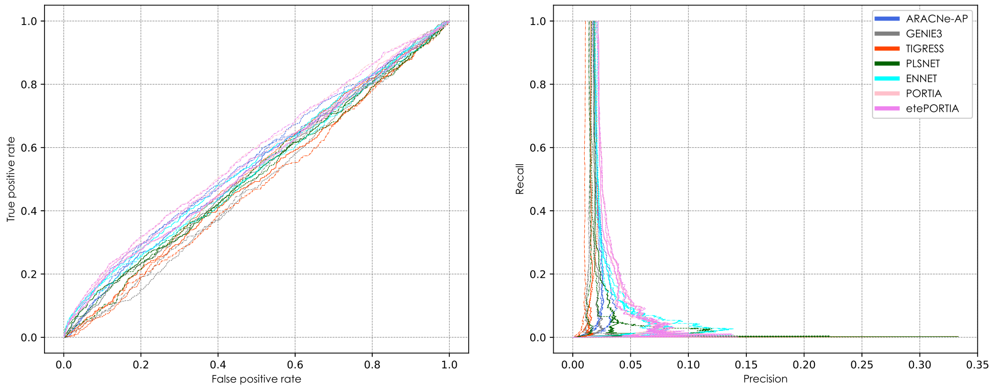


Figure S5: (Left) Receiver operating characteristic (Right) and Precision-recall curves of different GRN inferences methods on the StressResp expression data. Dense, dot-dashed and dashed lines correspond to the MacIsaac2, YEASTRACT Count3 and YEASTRACT Type2 goldstandard networks, respectively.

## S6.2 Effect of KO removal

In Tab. S13, S14 and S15, we reported the AUPR, AUROC and overall scores of the inferred networks, after removal of all KO experiments from the expression data. Because ENNET, PORTIA and etePORTIA explicitly model KO data through the calculation of Z-scores, these methods suffered from the strongest performance degradation, after KO removal. On DREAM3 and DREAM4, TIGRESS now outperforms the other methods, followed by ENNET, etePORTIA and PORTIA. PORTIAstill outperforms ARACNe-AP, GENIE3, PLSNET, despite being deprived from its only external source of directional information.

Table S13: AUROC, AUPR and overall scores of different GRN inference methods, evaluated on the 5 networks from DREAM3 (no KO experiment).

| Method | Net1 | | Net2 | | Net3 | | Net4 | | Net5 | | Overall score |
|---|---|---|---|---|---|---|---|---|---|---|---|
| | AUPR | AUROC | AUPR | AUROC | AUPR | AUROC | AUPR | AUROC | AUPR | AUROC | |
| ARACNe-AP | 0.020 | 0.562 | 0.028 | 0.552 | 0.036 | 0.566 | 0.054 | 0.524 | 0.062 | 0.506 | 2.475 |
| GENIE3 | 0.017 | 0.564 | 0.011 | 0.484 | 0.019 | 0.490 | 0.037 | 0.488 | 0.058 | 0.504 | 0.574 |
| PLSNET | 0.014 | 0.490 | 0.019 | 0.412 | 0.036 | 0.622 | 0.057 | 0.538 | 0.071 | 0.499 | 2.742 |
| TIGRESS | **0.049** | **0.758** | **0.051** | **0.693** | 0.045 | 0.629 | **0.065** | 0.560 | 0.071 | **0.530** | **8.128** |
| ENNET | 0.033 | 0.645 | 0.036 | 0.609 | **0.052** | **0.640** | 0.054 | 0.531 | **0.075** | 0.522 | 5.372 |
| PORTIA | 0.017 | 0.562 | 0.016 | 0.547 | 0.037 | 0.605 | 0.059 | 0.581 | 0.065 | 0.530 | 3.492 |
| etePORTIA | 0.017 | 0.576 | 0.017 | 0.554 | 0.039 | 0.608 | 0.062 | **0.585** | 0.065 | 0.529 | 3.791 |

Table S14: AUROC, AUPR and overall scores of different GRN inference methods, evaluated on the 5 networks from DREAM4 (with no KO experiment).

| Method | Net1 | | Net2 | | Net3 | | Net4 | | Net5 | | Overall score |
|---|---|---|---|---|---|---|---|---|---|---|---|
| | AUPR | AUROC | AUPR | AUROC | AUPR | AUROC | AUPR | AUROC | AUPR | AUROC | |
| ARACNe-AP | 0.049 | 0.621 | 0.061 | 0.584 | 0.096 | 0.632 | 0.059 | 0.611 | 0.079 | 0.648 | 10.086 |
| GENIE3 | 0.030 | 0.545 | 0.032 | 0.501 | 0.022 | 0.524 | 0.035 | 0.561 | 0.023 | 0.538 | 1.840 |
| PLSNET | 0.043 | 0.715 | 0.040 | 0.623 | 0.047 | 0.666 | 0.055 | 0.677 | 0.050 | 0.686 | 10.046 |
| TIGRESS | **0.091** | **0.808** | 0.072 | 0.692 | **0.161** | **0.800** | **0.099** | **0.750** | **0.108** | 0.767 | **24.873** |
| ENNET | 0.067 | 0.760 | **0.094** | **0.753** | 0.153 | 0.747 | 0.077 | 0.739 | 0.100 | **0.784** | 23.886 |
| PORTIA | 0.058 | 0.682 | 0.057 | 0.657 | 0.069 | 0.720 | 0.052 | 0.669 | 0.058 | 0.710 | 12.960 |
| etePORTIA | 0.067 | 0.726 | 0.060 | 0.666 | 0.078 | 0.718 | 0.070 | 0.676 | 0.078 | 0.726 | 15.397 |

Table S15: ROC-AUC scores of different GRN inference methods, evaluated on the 4 networks proposed in the DREAM5 GRN inference sub-challenge (no KO experiment).

| Method | Net1 | | Net3 | | Net4 | | Overall score |
|---|---|---|---|---|---|---|---|
| | AUPR | AUROC | AUPR | AUROC | AUPR | AUROC | |
| ARACNe-AP | 0.151 | 0.667 | 0.051 | 0.561 | 0.019 | 0.511 | 0.418 |
| GENIE3 | 0.016 | 0.530 | 0.014 | 0.498 | 0.017 | 0.493 | 0.000 |
| PLSNET | 0.227 | **0.846** | 0.052 | 0.572 | 0.022 | 0.518 | 34.251 |
| TIGRESS | 0.313 | 0.783 | 0.069 | 0.593 | 0.020 | 0.516 | 33.914 |
| ENNET | **0.379** | 0.832 | 0.053 | 0.598 | 0.021 | 0.517 | **65.948** |
| PORTIA | 0.316 | 0.766 | **0.117** | **0.614** | **0.027** | **0.532** | 41.691 |
| etePORTIA | 0.319 | 0.768 | **0.117** | 0.613 | **0.027** | **0.532** | 43.143 |

## S6.3 Symmetry of inferred adjacency matrices

In Fig. S6, S7, S8, S9 and S10, we reported the symmetry of networks inferred from each expression dataset considered in our study. For MERLIN+P, each yeast expression dataset has been evaluated three-fold, using different goldstandard networks. The LCL dataset was evaluated with two different goldstandard networks.

We first observe the inability of ARACNe-AP to infer asymmetric adjacency matrices. Indeed, the method is based on mutual information, which is a non-linear but symmetric correlation measure.

Symmetry of GRNs reconstructed by PORTIA seem to heavily depend on the presence of KO experiments. Indeed, symmetry is the highest on the DREAM4MF and MERLIN+P datasets, and inconsistent on DREAM5. Indeed, each gene expression dataset in DREAM5 has a variable number of available KO experiments. By simply dividing the number of KO experiments by the total number of genes, we obtain 2.1% (Net1), 0.86% (Net3) and 0.218% (Net4), respectively. The corresponding symmetry values have completely different orders of magnitude, that seem to confirm the strong improvement that is attributed to KO data. When only a few KO experiments are available, they appear to drastically improve the overall graph topology of inferred networks.

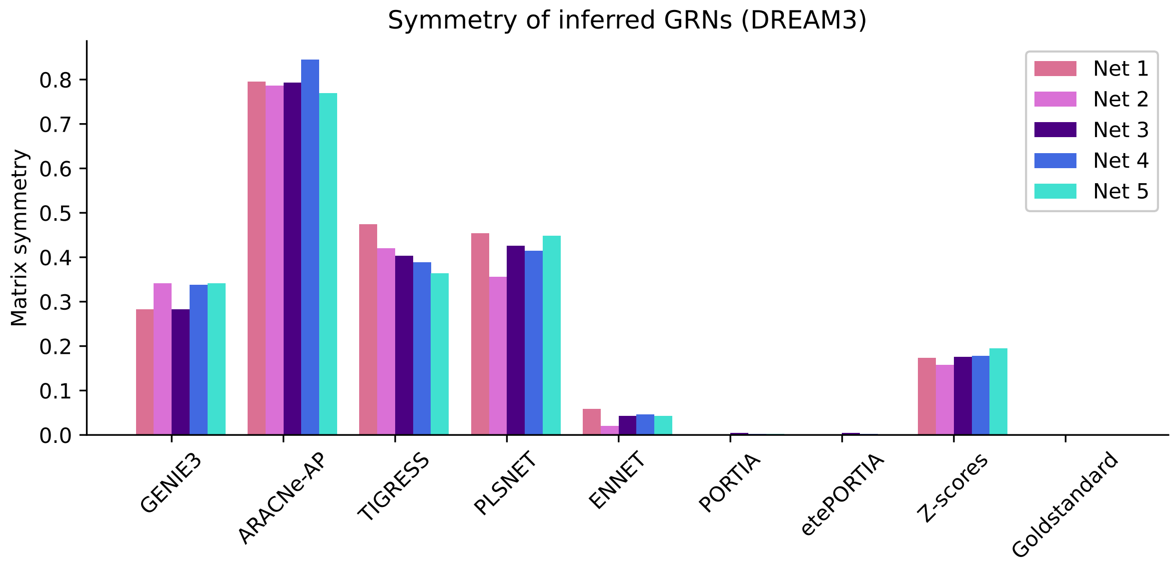


Figure S6: Symmetry of inferred networks (DREAM3).

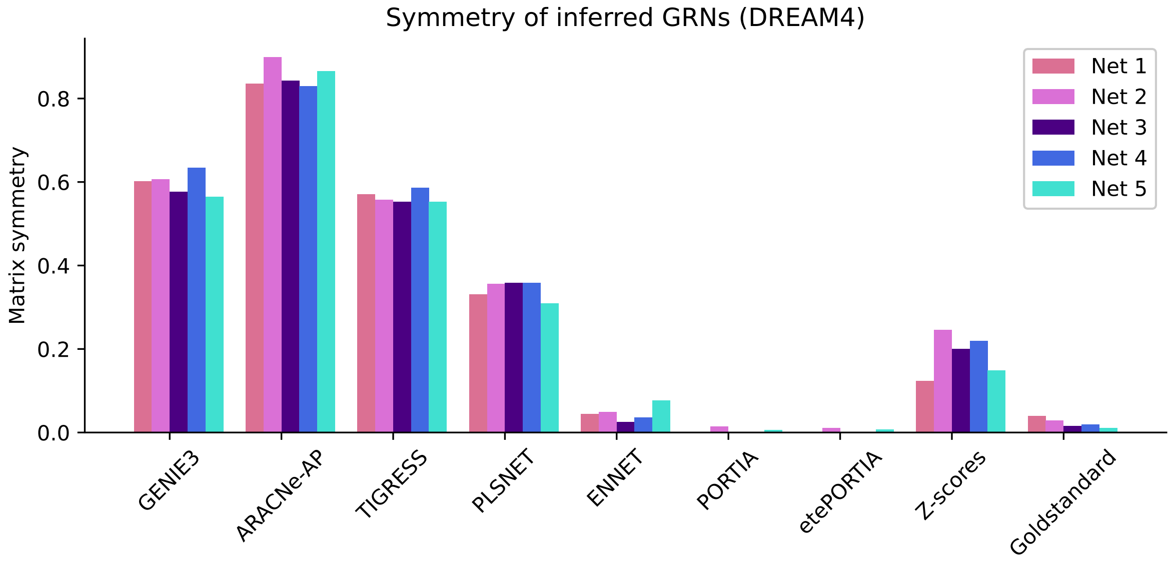


Figure S7: Symmetry of inferred networks (DREAM4).

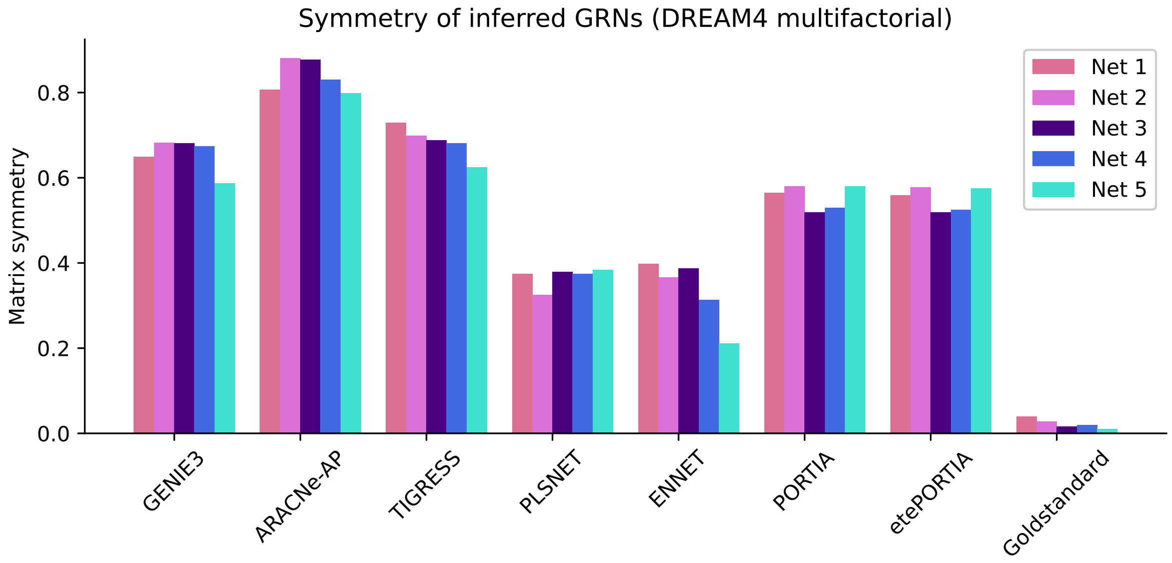


Figure S8: Symmetry of inferred networks (DREAM4MF)

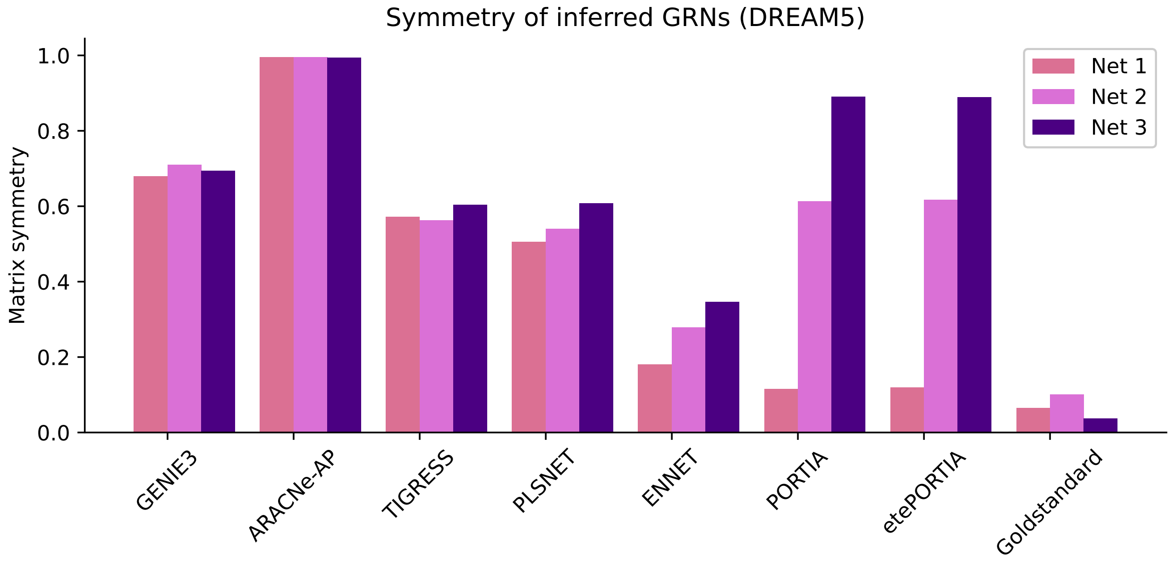


Figure S9: Symmetry of inferred networks (DREAM5)

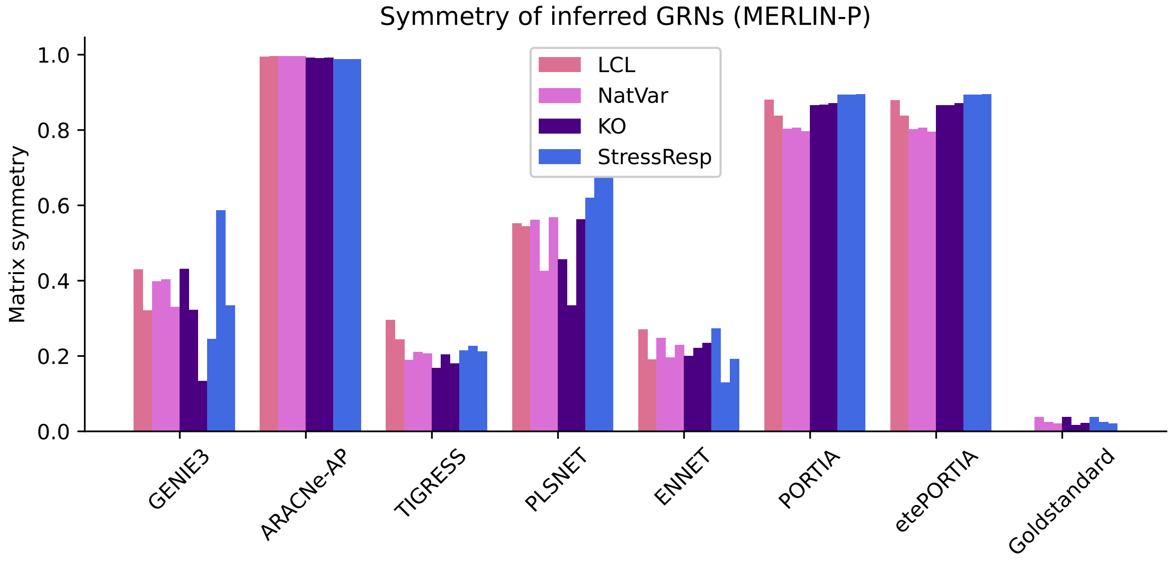


Figure S10: Symmetry of inferred networks (MERLIN+P)

# S7 Future work

One of the current limitations of our approach is the strong symmetry of inferred adjacency matrices in the absence of KO data, leaving room for future developments (e.g. L1 regularisation at the linear regression step, explicit modelling of time series, and a unified framework for time series, KO, and natural variation data). Another important improvement would be to find a new trick for introducing an intercept / bias term in the linear regressions without degrading the scalability of algorithm 1, for more accurate prediction of expression levels.

Moreover, the normality assumption of monotonically-transformed data might not be valid for all datasets, although it seems to be the case in the present context. In particular, GRN inference methods (including those relying on normality assumptions) have been reported to perform poorly on single-cell data [3, ]. Therefore, the generalisation of our approach to multimodal data could be a valuable development.

Finally, our simple model (PORTIA relies on a naive assumption of monotonicity) might also suggest that some of the recent advances in GRN inference might not be directly due to the sophistication of the nonlinear modelling, but on the good trade-off that feature selection-based methods offer between i) the ability to predict asymmetric GRN adjacency matrices, ii) the *implicit* quantification of the impact of KO experiments on TGs (e.g. GENIE3), and iii) the robustness to gene-specific biases (no crucial correction step is required).

# References


[1] George EP Box and David R Cox, *An analysis of transformations*, Journal of the Royal Statistical Society: Series B (Methodological) **26** (1964), no. 2, 211–243.

[2] Rachel B Brem and Leonid Kruglyak, *The landscape of genetic complexity across 5,700 gene expression traits in yeast*, Proceedings of the National Academy of Sciences **102** (2005), no. 5, 1572–1577.

[3] Shuonan Chen and Jessica C Mar, *Evaluating methods of inferring gene regulatory networks highlights their lack of performance for single cell gene expression data*, BMC bioinformatics **19** (2018), no. 1, 1–21.

[4] Gordon Chua, Quaid D Morris, Richelle Sopko, Mark D Robinson, Owen Ryan, Esther T Chan, Brendan J Frey, Brenda J Andrews, Charles Boone, and Timothy R Hughes, *Identifying transcription factor functions and targets by phenotypic activation*, Proceedings of the National Academy of Sciences **103** (2006), no. 32, 12045–12050.

[5] T Cokelaer, M Bansal, C Bare, E Bilal, BM Bot, E Chaibub Neto, F Eduati, M Gönen, SM Hill, B Hoff, JR Karr, R Küffner, MP Menden, P Meyer, R Norel, A Pratap, RJ Prill, MT Weirauch, JC Costello, G Stolovitzky, and J Saez-Rodriguez, *Dreamtools: a python package for scoring collaborative challenges [version 1; peer review: 3 approved with reservations]*, F1000Research **4** (2015), no. 1030.

[6] Darren A Cusanovich, Bryan Pavlovic, Jonathan K Pritchard, and Yoav Gilad, *The functional consequences of variation in transcription factor binding*, PLoS genetics **10** (2014), no. 3, e1004226.

[7] Jerome Friedman, Trevor Hastie, and Robert Tibshirani, *Sparse inverse covariance estimation with the graphical lasso*, Biostatistics **9** (2008), no. 3, 432–441.

[8] Zhanzhi Hu, Patrick J Killion, and Vishwanath R Iyer, *Genetic reconstruction of a functional transcriptional regulatory network*, Nature genetics **39** (2007), no. 5, 683–687.

[9] Diederik P Kingma and Jimmy Ba, *Adam: A method for stochastic optimization*, arXiv preprint arXiv:1412.6980 (2014).

[10] Tuuli Lappalainen, Michael Sammeth, Marc R Friedländer, Peter Ac‘t Hoen, Jean Monlong, Manuel A Rivas, Mar Gonzalez-Porta, Natalja Kurbatova, Thasso Griebel, Pedro G Ferreira, et al., *Transcriptome and genome sequencing uncovers functional variation in humans*, Nature **501** (2013), no. 7468, 506–511.

[11] Daniel Marbach, James C Costello, Robert Küffner, Nicole M Vega, Robert J Prill, Diogo M Camacho, Kyle R Allison, Manolis Kellis, James J Collins, and Gustavo Stolovitzky, *Wisdom of crowds for robust gene network inference*, Nature methods **9** (2012), no. 8, 796–804.

[12] Nifang Niu, Yuxin Qin, Brooke L Fridley, Junmei Hou, Krishna R Kalari, Minjia Zhu, Tse-Yu Wu, Gregory D Jenkins, Anthony Batzler, and Liewei Wang, *Radia-*

*tion pharmacogenomics: a genome-wide association approach to identify radiation response biomarkers using human lymphoblastoid cell lines*, Genome research **20** (2010), no. 11, 1482–1492.

[13] Adam Paszke, Sam Gross, Francisco Massa, Adam Lerer, James Bradbury, Gregory Chanan, Trevor Killeen, Zeming Lin, Natalia Gimelshein, Luca Antiga, et al., *Pytorch: An imperative style, high-performance deep learning library*, Advances in neural information processing systems **32** (2019).

[14] Thomas Schaffter, Daniel Marbach, and Dario Floreano, *Genenetweaver: in silico benchmark generation and performance profiling of network inference methods*, Bioinformatics **27** (2011), no. 16, 2263–2270.

[15] Erin N Smith and Leonid Kruglyak, *Gene–environment interaction in yeast gene expression*, PLoS biology **6** (2008), no. 4, e83.

[16] Jun Zhu, Pavel Sova, Qiuwei Xu, Kenneth M Dombek, Ethan Y Xu, Heather Vu, Zhidong Tu, Rachel B Brem, Roger E Bumgarner, and Eric E Schadt, *Stitching together multiple data dimensions reveals interacting metabolomic and transcriptomic networks that modulate cell regulation*, PLoS biology **10** (2012), no. 4, e1001301.
